# Bidirectional Soft Catalysts for Improved Power Performance of Li‐S Batteries

**DOI:** 10.1002/advs.77302

**Published:** 2026-08-21

**Authors:** Maleesha M. Nishshanke, Petar Jovanovic, Swarit Dwivedi, Declan McNamara, Akshat Tanksale, Mahdokht Shaibani, Matthew Hill, Mainak Majumder

**Affiliations:** ^1^ Nanoscale Science and Engineering Laboratory (NSEL) Department of Mechanical and Aerospace Engineering Monash University Melbourne VIC Australia; ^2^ ARC Research Hub For Advanced Manufacturing With 2D Materials Monash University Melbourne VIC Australia; ^3^ Department of Materials Science and Engineering Monash University Melbourne VIC Australia; ^4^ Department of Chemical & Biological Engineering Monash University Melbourne VIC Australia; ^5^ Department of Chemical and Environmental Engineering RMIT University Melbourne VIC Australia

**Keywords:** high‐power pouch‐cells, lithium‐sulfur battery, metal catalysts, microenvironment engineering, polymer‐metal complexes

## Abstract

Lithium‐sulfur (Li‐S) batteries are attractive for next‐generation energy storage owing to their high theoretical energy density and low cost, yet their practical performance is limited by sluggish redox kinetics. Here, we introduce a soft catalyst in which a polymer‐metal complex is directly integrated into the binder, creating a catalytically active microenvironment that enables efficient and bidirectional sulfur catalysis. A polyvinyl alcohol (PVA) scaffold stabilizes metal ions through chelation with the electron‐donating ‐OH groups of PVA, forming homogeneously distributed catalytic domains in the sulfur cathode. Among the systems investigated, PVA‐Cu exhibits the highest catalytic activity, arising from optimal sulfur binding energies, Cu‐S d‐p hybridization, and additional electronic states near the Fermi level. As a result, the PVA‐Cu cells deliver high capacities at 3 mg cm^−2^ of 1,100 mAh g^−1^ at 1 C with 82% capacity retention over 300 cycles, while at higher loadings of 9 mg cm^−2^, the cells deliver 813 mAh g^−1^ (7.3 mAh cm^−2^) at 0.5 C. In a pouch cell prototype, stable cycling at 0.5 C delivers a discharge capacity of 790 mAh g^−1^ and a power density of 150 W kg^−1^, which lies within the upper range of reported Li‐S pouch‐cell performance in terms of operating rate and power density.

## Graphical abstract

A soft polymer‐metal catalyst is integrated into the binder to create a catalytically active microenvironment that accelerates bidirectional sulfur redox kinetics in lithium‐sulfur batteries. A model polyvinyl alcohol‐Cu catalyst enables high areal capacities at demanding current densities and stable cycling in pouch‐cell prototypes at 0.5 C, achieving power densities of 130–150 W kg^−1^.

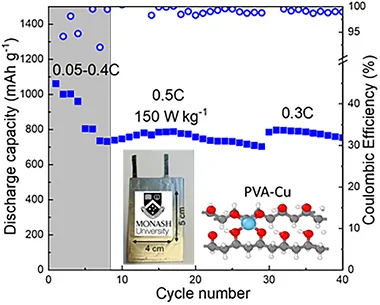

## 1 Introduction

As the world transitions toward carbon neutrality, the need for high‐performance, sustainable and low‐cost energy storage solutions has never been more pressing [1]. Li‐S batteries have emerged as a promising next‐generation energy storage technology owing to their high theoretical capacity (1,675 mAh g^−1^) and gravimetric energy density (2,600 Wh kg^−1^), offering the potential for lighter and longer‐lasting energy storage for electric mobility, aerospace and portable electronics [2]. As an active material, sulfur has a compelling advantage because it is earth‐abundant, low‐cost and environmentally benign [3].

At the heart of this promise lies a complex multi‐electron, multi‐phase reaction. In conventional ether electrolytes, sulfur undergoes a stepwise reduction reaction from solid S_8_ to soluble lithium polysulfide (LiPS) intermediates (Li_2_S_x_, 4 ≤ x ≤ 8), followed by a rate‐limiting liquid‐solid phase transition to Li_2_S, and the reverse in oxidation [4]. The 16‐electron electrochemical conversion enables a very high capacity, but poses two major challenges.

Under concentration gradients, the soluble LiPS readily diffuse toward the lithium anode, where they corrode the surface and induce a continual loss of active material, known as the “shuttle effect” [5]. In addition, sulfur redox processes are plagued by sluggish kinetics, particularly during the nucleation and growth of solid‐phase Li_2_S [6]. The insulating nature of Li_2_S and its tendency to precipitate as film‐like, passivating deposits hinder ion and electron transport, limiting active material utilization [7]. The root cause of these challenges lies in the weak adsorption and low interfacial charge transfer efficiency between sulfur species and the conventional carbon hosts. As a result, sulfur cathodes inherently suffer from poor rate performance and rapid capacity decay, limiting their practical performance [8].

Over the past decade, numerous strategies have been devised to address these challenges, most notably through catalysis. Catalytic materials have been introduced into the cathode, electrolyte or separator [4, 5, 9, 10, 11, 12, 13, 14, 15], playing several crucial roles. Namely, (1) immobilizing sulfur species through physical or chemical adsorption, (2) reducing the activation energy barriers to promote charge transfer [16] and (3) offering an energetically favourable interface for Li_2_S deposition and growth [17]. Among these, transition metal‐based catalysts (e.g. Co, Ni, Fe, Zn and Cu) have proven particularly effective [3, 18, 19], leveraging their partially filled d‐orbitals to lower activation barriers and stabilize transition states through favourable metal‐sulfur binding interactions. Sophisticated architectures ranging from nanoparticles [20] to single atom catalysts [21], oxides [22], sulfides [3], selenides [23], defect‐engineering [24], and heterostructuring [25, 26] have pushed the frontiers of performance by modulating d‐band centres and optimizing binding electron transport dynamics [27, 28].

Despite these advances, many catalytic systems still face issues of limited site accessibility, passivation, agglomeration and degradation, reducing their catalytic efficiency and longevity [29, 30, 31]. This becomes particularly pronounced under practical or high sulfur loadings where shuttling and mass transport limitations dominate, further exacerbating the already poor kinetics [32, 33]. As a result, most studies rely on low sulfur loadings (<2 mg cm^−2^), overestimating rate performance. As a result, there is a persistent gap in demonstrating higher cycling rates (>1 C) at practically relevant loadings (≥3 mg cm^−2^).

An emerging perspective from the catalysis community is shifting attention from the rational design of the active sites [34] toward engineering the broader microenvironment surrounding them [35, 36]. Recent advances increasingly embrace on the concept of soft catalysis, where the active site is coordinated or anchored within a soft matter, an adaptable and dynamic matrix such as polymers or supramolecular assemblies that actively regulate the local catalytic microenvironment rather than rigid catalytic frameworks [37]. Among these architectures, polymer‐metal coordination complexes, as soft catalysts, combine the intrinsic reactivity of metal centers with the structural versatility afforded by polymer frameworks, yielding dynamic reaction environments with abundant and highly accessible active sites [38, 39, 40, 41, 42]. The polymer microenvironment has the potential to influence electron transfer and mass transport in its immediate surroundings, thereby modulating the electronic structure and charge distribution [40, 43, 44]. In nature, enzymes provide a compelling analogue that exemplifies this principle. Their microenvironments exert precise control over binding energies, charge distribution and reaction pathways with remarkable efficiency and selectivity [45].

Within the broader family of coordination polymers, metal–organic frameworks (MOFs), have attracted significant attention in Li‐S batteries for their tunable pore structures, high surface areas, and well‐defined, abundant catalytic sites [46]. However, MOFs are crystalline coordination networks that often suffer from structural instability and poor scalability [47, 48]. In contrast, amorphous polymer‐metal coordination systems, as explored in this work, provide a promising alternative that offers scalable and facile synthesis, improved stability and dynamically accessible catalytic environments [48]. The present work establishes polymer‐metal coordination as a broader catalytic platform by extending the concept of soft and dynamic catalytic microenvironments to sulfur redox chemistry. We demonstrate that tailoring the catalytic microenvironment governs both sulfur reduction and oxidation kinetics, enabling high‐rate and practical battery performance.

Motivated by the need to achieve efficient bidirectional catalysis without the reliance on complex architectures or synthesis procedures, we introduce a model polymer‐metal soft catalyst that is directly integrated into the binder of the sulfur cathode. In our design, a polyvinyl alcohol (PVA) scaffold is coordinated with metal ions (Cu^2+^, Fe^3+^, Zn^2+^, Ga^3+^ and Al^3+^), forming electronically active catalytic domains that are homogeneously dispersed within the electrode.

Among these, the PVA‐Cu complex exhibits the most pronounced enhancement in both the reduction and oxidation kinetics. Density functional theory (DFT) calculations reveal that this is made possible by a favourable thermodynamic and electronic landscape. The polar PVA‐Cu microenvironment offers favourable binding and stabilization on LiPS and Li_2_S and suppresses shuttling, while simultaneously introducing additional states near the Fermi level. The strong hybridization between the Cu d‐orbitals and S‐p orbitals enhances local electronic delocalization, narrows interfacial band gaps and lowers kinetic barriers to sustain a continuous and efficient catalytic cycle across all sulfur conversion reactions.

When integrated into Li‐S cells at practical sulfur loadings (3–9 mg cm^−2^), the polymer‐metal soft catalysts exhibit outstanding electrochemical performance. At 3 mg cm^−2^ and 1 C, the PVA‐Cu cathode delivers an initial capacity of 1,100 mAh g^−1^ with 82% retention over 300 cycles. At a higher loading of 6 mg cm^−2^ and at 1 C, the cell achieves a capacity of 622 mAh g^−1^ (3.7 mAh cm^−2^) with 70% capacity retention after 100 cycles. Even at 9 mg cm^−2^, the cells attain an initial discharge capacity of 813 mAh g^−1^ (7.3 mAh cm^−2^) at 0.5 C.

Crucially, the PVA‐Cu cathodes achieve high areal capacities at demanding current densities (5–10 mA cm^−2^), demonstrating the ability of the soft catalyst microenvironment to reconcile high‐rate operation with practical sulfur loadings. When translated to a pouch cell prototypes, we achieve stable cycling at 0.5 C with a power densities of 130–150 W kg^−1^. These results position PVA‐Cu among the limited number of reported Li‐S pouch cell studies capable of operating at such high rates while maintaining practical power densities.

## 2 Results and Discussion

### 2.1 Synthesis and Characterization of Polymer‐Metal Complexes

PVA is a non‐toxic, water‐soluble, and semicrystalline polymer that can adsorb, reduce, and stabilise metal cations [49]. The polymer‐metal hybrids are synthesised by complexing PVA with divalent and trivalent metal cations, including Cu^2+^, Fe^3+^, Zn^2+^, Ga^3+^ and Al^3+^ in an aqueous medium to form PVA‐Cu, PVA‐Fe, PVA‐Zn, PVA‐Ga, and PVA‐Al. Lewis acid metal ions undergo chelation with the electron‐donating ‐OH groups of PVA, forming coordination bonds through an acceptor‐donor mechanism, consistent with previous reports of polymer‐metal complexes such as PVA‐Cu, Fe, Al, Mg, Zr [50, 51, 52]. The structures of the different complexes are shown in Figure 1A and Figure S1, based on reactive molecular dynamics simulations. Two protons are removed from the PVA double layer and replaced with divalent metal cations (Cu^2+^, Zn^2+^) to form charge‐neutral divalent PVA‐metal complexes. Similarly, three protons are removed to form trivalent PVA complexes with Fe^3+^, Ga^3+^ and Al^3+^. The simulations indicate that their formation is thermodynamically favourable, as evidenced by the calculated negative formation energies (Figure 1B).

**FIGURE 1:**
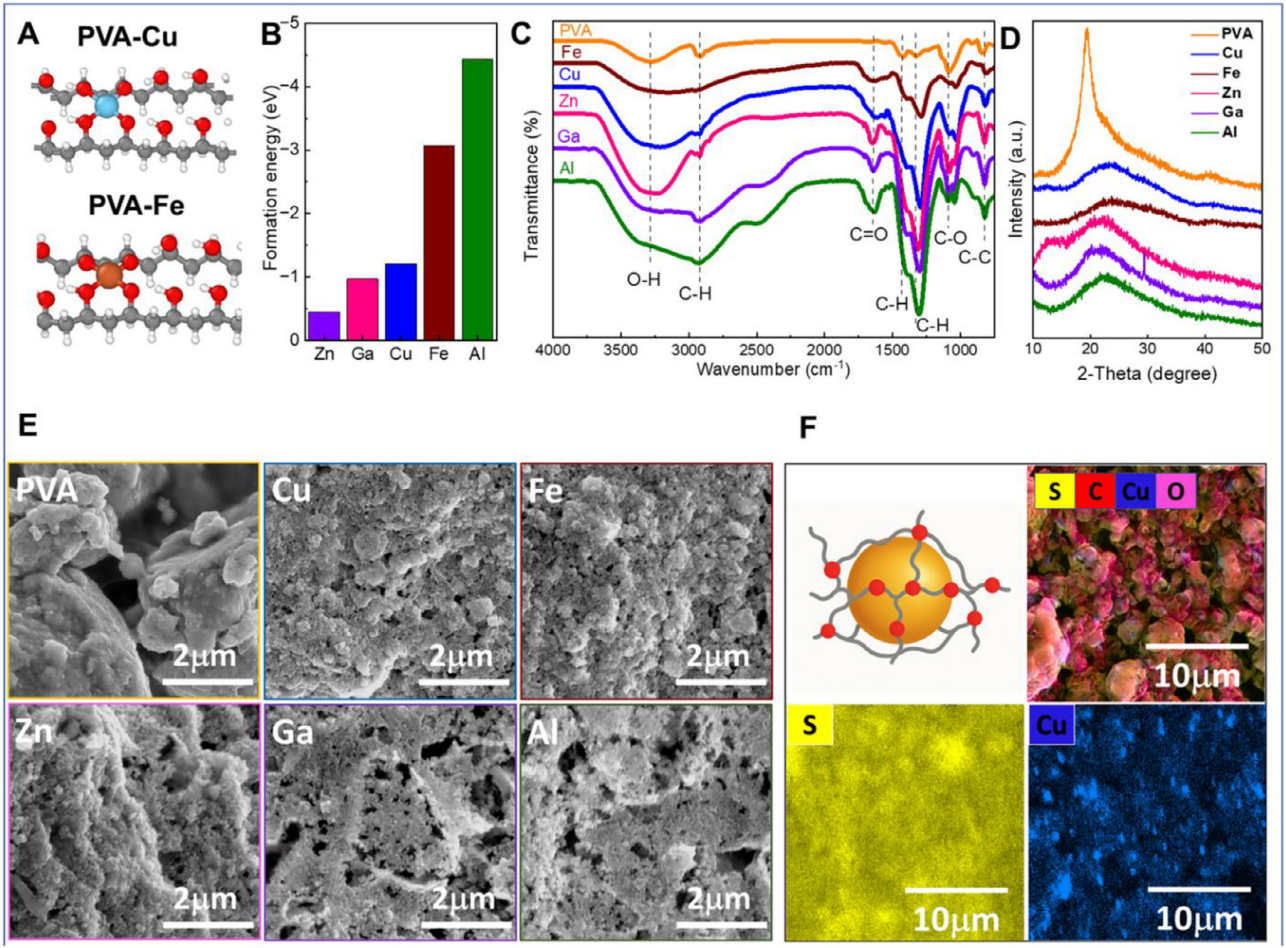
Polymer‐metal hybrid catalysts design and characterization. (A) Molecular configurations of PVA‐Cu and PVA‐Fe polymer‐metal complexes, representing the formation of polymer‐metal complexes between PVA with divalent and trivalent metal cations. (B) The formation of various PVA‐metal coordination complexes is confirmed by thermodynamically favourable formation energies, which are calculated theoretically. (C) Shifted wavenumbers at different peaks in the FTIR spectra and (D) a decrease in the PVA crystalline peak in the XRD confirm the PVA‐metal cation interactions experimentally. (E,F) Microstructural study of sulfur cathodes having PVA‐metal catalytic binders. (E) The top‐view SEM images highlight the differences between the microstructure of pristine PVA cathodes and that of PVA‐metal cathodes. (F) The sulfur is encapsulated by PVA‐metal binders, forming a cohesive and integrated structure: Schematic illustration and elemental mapping of homogeneous Cu distribution in the PVA‐Cu cathodes.

Experimental characterization further corroborates the formation of coordination complexes. From Figure 1c, the Fourier Transform Infrared spectroscopy (FTIR) spectra exhibit a broadening of the O─H stretching vibration around 3500 cm^−1^, and shifts in the C═O carbonyl stretch at 1690 cm^−1^ and C─H bending and deformation stretching regions at 1425 and 1324 cm^−1^, respectively, indicative of interactions between the metal ions and the hydroxyl/carbonyl groups of PVA, in line with previous observations in the literature [49, 50]. The X‐ray Diffraction (XRD) pattern in Figure 1d reveals that the strong diffraction peak around 2θ = 19.4° of PVA, corresponding to the (101) crystal plane [49, 50], becomes weaker and slightly shifted after complexation with metal cations. This reduction in crystallinity arises because coordination between metal ions and PVA chains disrupts interchain hydrogen bonding between ─OH groups and reduces the crystallinity of PVA [52]. Transmission electron microscopy‐based energy‐dispersive X‐ray (TEM‐EDX) mapping of the PVA‐Cu complex (Figure S2) demonstrates a homogeneous distribution of Cu throughout the polymer matrix, confirming the well‐dispersed nature of catalytic metal centers within the polymer‐metal system.

Aligned with standard electrode fabrication methods, sulfur cathodes are fabricated by dispersing an aqueous slurry consisting of 65 wt.% sulfur, 20 wt.% conductive carbon, and 15 wt.% binder system that includes PVA‐metal complexes and carboxymethylcellulose (CMC) as the structural polymer to withstand the volume expansion, while the PVA‐metal complexes both bind the electrode components and actively participate in sulfur redox chemistry. We fabricate six different types of sulfur cathode, including various PVA‐metal hybrids in the binder system and a control sample without any metals in the binder system. The composition of each cathode is listed in Table S1.

Scanning electron microscopy (SEM) images of the cathodes with PVA and PVA‐metal (Cu, Fe, Zn, Ga, and Al) binder systems illustrated in Figure 1e and Figure S3. The PVA control cathode exhibits some areas of particle aggregation and polymer coverage, expected for polymer‐only binder systems [53], although on the whole, the microstructure displays a reasonably porous structure. The PVA‐metal hybrid cathodes show a more uniform binder distribution and improved structural integrity. Specifically, the PVA‐Cu, PVA‐Fe and PVA‐Zn cathodes present more compact morphologies composed of fine, discrete particles, whereas the PVA‐Ga and PVA‐Al cathodes feature similarly compact but moderately porous microstructures. Despite the apparent differences in morphology, the ion‐transport resistance, porosity, and tortuosity remain comparable between the PVA and PVA‐Cu electrodes (8.2 Ω cm^2^, 61.7%, 4.6 for PVA‐Cu vs. 11.9 Ω cm^2^, 63.8%, 6.8 for PVA, respectively) (Table S2 and Figure S4). The modest reduction in tortuosity (∼2%) and ion‐transport resistance (∼4 Ω cm^2)^ in PVA‐Cu compared to PVA suggests the presence of low‐tortuosity ion‐transport pathways and enhanced ion‐transfer kinetics within the cathode. However, given the relatively small magnitude of these differences, the influence of electrode microstructural factors, such as Li^+^ transport and pore characteristics, can be decoupled from the observed electrochemical performance.

As illustrated schematically in Figure 1f, the polymer‐metal complexes encapsulate the sulfur particles, indicating the structural role of the PVA‐metal binder in establishing a well‐integrated cathode architecture with uniformly distributed catalytic sites. This is supported by EDX mapping (Figure 1f and Figure S5), which reveals a homogeneous distribution of the PVA‐metal binders and uniform dispersion of metal centres across the sulfur particles within the electrode matrix.

### 2.2 Efficient Reduction and Oxidation Enabled by Polymer‐metal Complexes

At a practical sulfur loading of 3 mg cm^−2^ and at 1 C (Figure 2a), all PVA‐metal hybrid cathodes deliver higher discharge capacities than the PVA‐only control. Among these, the PVA‐Cu system exhibits the highest average specific capacity of 1,130 mAh g^−1^, followed by PVA‐Fe (992 mAh g^−1^), PVA‐Zn (983 mAh g^−1^), PVA‐Ga (895 mAh g^−1^) and PVA‐Al (837 mAh g^−1^), while the control PVA only cell delivers only 745 mAh g^−1^.

**FIGURE 2:**
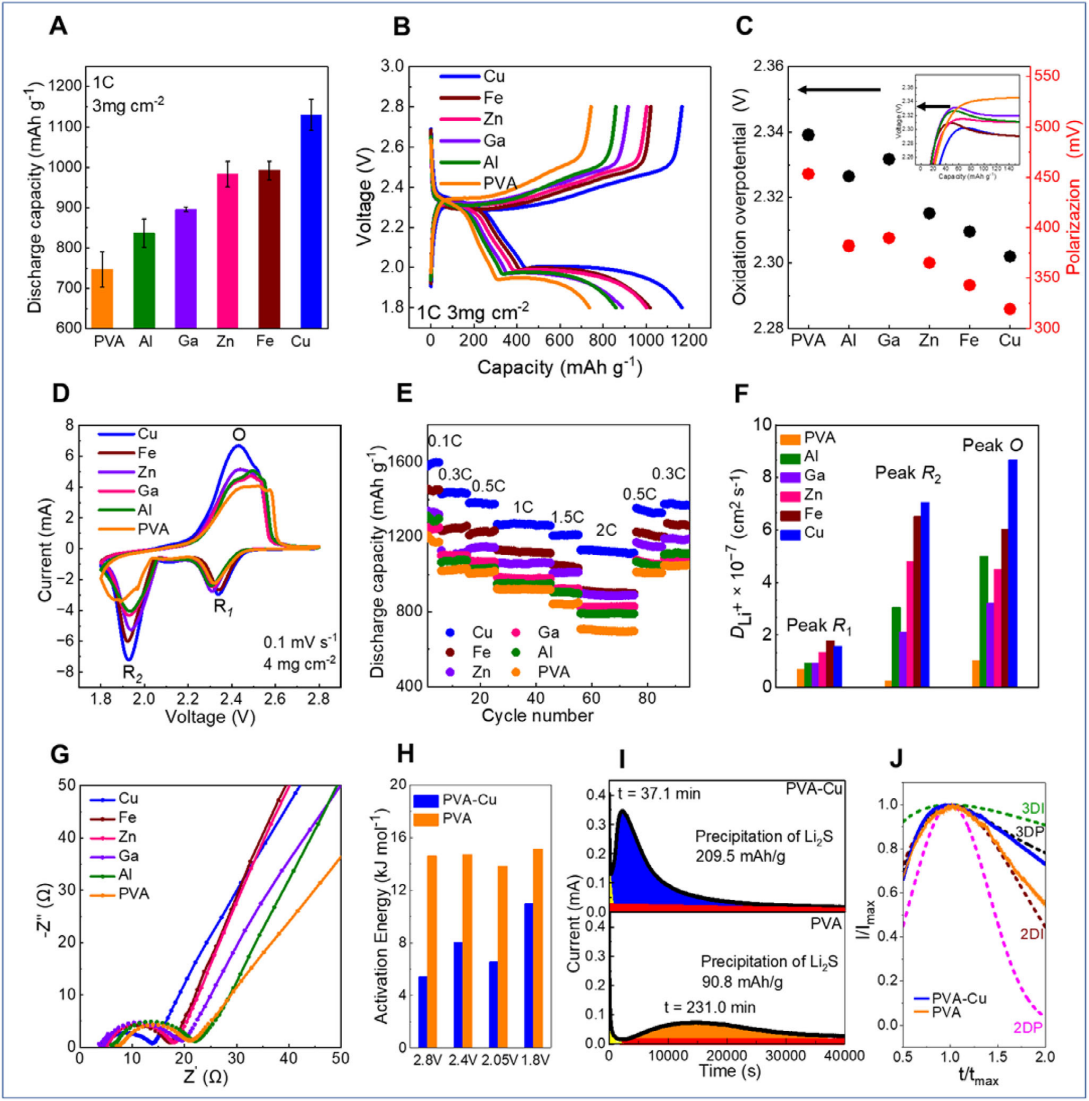
Cycling and electrochemical performance by PVA‐metal catalytic binders in Li‐S batteries. (A) A comparison of discharge capacities at sulfur loading of 3 mg cm^−^^2^ at 1 C demonstrates PVA‐metal cathodes deliver higher capacities (The reported values represent averages from three independently assembled cells, with error bars indicating the standard deviation). (B) PVA‐metal cathodes exhibit extended discharge plateaus in the galvanostatic charge‐discharge curves under identical operating conditions, indicating enhanced electrochemical performance. (C) The PVA‐metal binders contribute to a reduction in both polarisation and oxidation overpotentials, where PVA‐Cu exhibits the lowest. (D) Enhanced CV peak currents in liquid‐solid‐liquid phase reactions by PVA‐metal catalysts. (E) The rate capability of PVA‐metal and PVA cathode systems shows better performance of PVA‐Cu across current densities from 0.1 to 2 C. (F) The *D*_Li_^+^ values in PVA‐metal cathodes (G) Nyquist plots of Li‐S batteries showing a decrease in *R*_ct_ values with PVA‐metal catalysts. (H) Activation energies of PVA‐Cu and PVA cathodes at various discharge voltages, demonstrating the low energy required for Li‐S redox reactions in the PVA‐Cu system. (I) Potentiostatic discharge profiles of Li_2_S_8_ cells containing PVA‐Cu and PVA reveal that the PVA‐Cu system attains a higher Li_2_S deposition capacity in a shorter time. (J) Current‐time profiles of Li_2_S deposition on PVA‐Cu and PVA cathodes, showing evidence of three‐dimensional Li_2_S growth on the PVA‐Cu surface.

Representative galvanostatic charge‐discharge (GCD) profiles (Figure 2b) at 1 C reveal the characteristic two‐plateau discharge behaviour of Li‐S chemistry. The upper plateau near 2.4 V corresponds to the formation of long‐chain LiPS (Li_2_S_8_), followed by a ramp until 2.1 V, associated with the formation of Li_2_S_6_ and Li_2_S_4,_ and accounting for ∼25% of the capacity. The lower plateau represents the subsequent liquid‐solid and solid‐solid conversion from LiPS to Li_2_S_2_/Li_2_S, accounting for the remaining ∼75% of the theoretical capacity [54]. All PVA‐metal binders deliver extended discharge plateaus compared to the PVA control, indicating more complete conversions and accelerated reaction kinetics. In particular, the PVA‐Cu cells exhibit the longest and most stable plateaus, closely followed by PVA‐Fe and PVA‐Zn.

The charge‐discharge polarisation (ΔV) extracted from the GCD profiles (Figure 2c) captures the kinetics differences. Polarisation, defined as the deviation between the observed and equilibrium potentials, reflects the cumulative charge transfer, ion diffusion and ohmic resistances [55, 56]. Incorporating the PVA‐metal catalysts substantially reduces polarisation, from 453 mV in the PVA control to 319 mV in the PVA‐Cu cell, demonstrating more efficient charge transfer and reduced overpotentials. In addition, the voltage dip at the beginning of the second discharge plateau marks the onset of Li_2_S_2_/Li_2_S formation, and represents the initial nucleation barrier in this liquid‐solid transition [57]. The reverse process, Li_2_S_2_/Li_2_S dissolution, is marked by a sharp voltage rise at the start of the charging process, and represents the oxidation barrier [58]. These barriers are significantly reduced in PVA‐metal cathodes, suggesting that the polymer‐metal complexes enable a more favourable three‐phase interface between the electrolyte, catalyst and conductive network that reduces the energy barrier to nucleation, growth and subsequent dissolution of Li_2_S [59].

Cyclic voltammetry (CV) at 0.1 mV s^−1^ (Figure 2d) corroborates these insights. Two cathodic peaks appear at ∼2.4 V (*R*_1_, LiPS formation and interconversion) and at ∼2.0 V (*R*_2_, Li_2_S formation), while a broad anodic peak at ∼2.4 V (*O*) corresponds to the reverse process. PVA‐metal binders exhibit higher peak currents than the PVA control. PVA‐Cu again stands out, showing the highest cathodic and anodic currents and the lowest polarization (Figure S6), confirming its strong catalytic influence on both liquid‐liquid and liquid‐solid conversion reactions. The corresponding Tafel slopes in Figure S7, an indicator of kinetics, further reinforce this. The PVA‐Cu, Fe and Zn yield slopes of 56, 59 and 64 mV dec^−1^ for R_2_, respectively, are far lower than PVA at 91 mV dec^−1^.

Rate capability tests confirm that the PVA‐metal catalysts improve sulfur utilization and redox kinetics, outperforming PVA‐only cells at rates from 0.1 C and up to 2 C (Figure 2e). This reflects suppressed LiPS shuttling, enhanced ion transport and faster redox kinetics. PVA‐Cu simultaneously achieves high capacity at low rates (1,598 mAh g^−1^ at 0.1 C) and at high rates (1,131 mAh g^−1^ at 2 C), which translates to 71% retention from 0.1 to 2 C. In contrast, the PVA‐only cell attains lower capacities (1,208 mAh g^−1^ at 0.1 C and 708 mAh g^−1^ at 2 C) with a poorer retention of 58%. This corresponds to sulfur utilization ranging from 94% to 67% for PVA‐Cu at 0.1 and 2 C, respectively, compared with 72% to 42% for the PVA‐only system (Figure S8). The performance hierarchy in terms of capacity at 2 C (PVA‐Cu >PVA‐Fe ≈ PVA‐Zn >PVA‐Ga ≈ PVA‐Al >PVA) confirms that transition‐metal catalysts (Fe, Cu and Zn) are the most efficient, while also demonstrating that even p‐block metals such as Ga and Al can contribute through their strong Lewis acid catalytic behaviour [60].

The Li^+^ diffusion coefficients (*D*_Li_^+^) are experimentally determined from scan‐rate‐dependent CVs in (Figure S9) using the Randles‐Sevcik relation [53]. The summarised results in Figure 2f show that strikingly, the PVA‐Cu binder yields enhanced *D*_Li_^+^ values of 1.6 × 10^−7^, 7.0 × 10^−7^ and 8.7 × 10^−7^ cm^2^ s^−1^ corresponding to the *R*_1_, *R*_2_ and *O* peaks, in contrast to PVA, which has the lowest *D*_Li_^+^ values of 0.7 × 10^−7^, 0.2 × 10^−7^ and 1.0 × 10^−7^ cm^2^ s^−1^ for the same peaks. This enhancement is particularly significant for the rate‐limiting liquid‐solid reaction peak *R*_2_, which increases by a factor of ∼35 compared with PVA. Given the first‐order dependence of sulfur redox kinetics, holding all else constant, the kinetics are strongly dependent on the local Li^+^ availability and electronic conductivity of sulfur [61, 62]. The higher *D*_Li_^+^ indicates faster ion transport and improved electronic properties in PVA‐Cu, which enable improved lithiation/ de‐lithiation dynamics. This is complemented with Galvanostatic Intermittent Titration Technique (GITT) in Figure S10, showing that at all states of discharge and charge, PVA‐Cu displayed a notably higher *D*_Li_^+^ compared to PVA cells, particularly at the second discharge plateau.

Electrochemical impedance spectroscopy (EIS) is conducted after several activation cycles (Figure 2g). The Nyquist plots are comprised of a high‐frequency semicircle corresponding to the charge transfer resistance (*R*_ct_) and a sloping straight line at lower frequencies related to ion diffusion [63]. The steeper slopes for PVP‐metal systems in the low‐frequency region also align with the higher *D*_Li_^+^ of these systems. Among the cells, PVA‐Cu exhibited the lowest *R*_ct_ of 9.5 Ω, while PVA exhibited a higher value of 15.3 Ω, respectively. The notably lower *R*_ct_ arises from the enhanced charge‐transfer [64] of PVP‐Cu and is in agreement with the lower polarization and improved rate capability. These differences can also be explained by the differences in electrical conductivity of the binder, holding all else constant, as the complexes show a consistently lower sheet resistance (Table S3). For example, the PVA‐Cu cathode exhibits the lowest sheet resistance (10.6 ± 1.5 Ω sq^−1^), compared to the PVA cathode (95.2 ± 9.8 Ω sq^−1^). Similar conductivity enhancements have been reported in polymer‐metal complexes, where coordination interactions reduce polymer crystallinity and increase the amorphous phase fraction to facilitate charge transport [65, 66, 67].

Temperature‐dependent EIS measurements across different discharge depths (2.8, 2.4, 2.05 and 1.8 V) further quantify reaction kinetics. The activation energy (*E*_a_) is derived from the Arrhenius relationship *R*_ct_ = A·e^(−^ *^E^* ^a/RT^), where *R* is the gas constant, *A* is the pre‐exponential factor, and *T* is the temperature [68, 69, 70]. The gradient of the ln(*R*) vs. 1/T plot represents the activation energy associated with the electrochemical reactions (Figure S11). Critically, these experiments reveal that PVA‐Cu exhibits a notably lower *E*_a_, particularly at voltages associated with liquid‐solid and solid‐solid conversions, reducing *E*_a_ from 13.8 to 6.5 kJ mol^−1^ at 2.05 V and from 15.2 to 11.0 kJ mol^−1^ at 1.8 V (Figure 2h).

To further elucidate and explicitly demonstrate how the polymer‐metal complexes (particularly PVA‐Cu) promote redox kinetics, symmetric‐cell tests are performed with Li_2_S_6_ and Li_2_S_8_ catholytes to decouple the liquid‐liquid and liquid‐solid conversion processes. From Figure S12a,b, the PVA‐Cu symmetric cells exhibit significantly higher redox peak currents and reduced impedance. Faster conversion kinetics shorten the residence time of soluble LiPS species in the electrolyte and are crucial for suppressing the shuttle effect. The Li_2_S deposition behaviour is then examined by the potentiostatic intermittent titration technique (PITT) in Figure 2i. The responses of the potentiostatic electrodeposition feature a sudden increase in the current response, a process characterized by the formation of isolated nuclei on the cathode substrate by overcoming the interfacial impedance between the electrolyte and the substrate, up to a maximum (I_max_) at a given time (t_max_). This is followed by a decrease in current, which is ascribed to the impingement and overlapping of nearby growing nuclei, and marks diminishing available interfaces, before terminating [7]. A shorter t_max_ reflects faster deposition and growth kinetics. The PVA‐Cu cell exhibits a notably higher deposition capacity with a sharper nucleation peak and a faster response (210 mAh g^−1^ and 37.1 min) compared to the PVA‐only cell (91 mAh g^−1^ and 231.0 min). The same observation is true for the oxidation step, with a Li_2_S dissolution capacity of 708 mAh g^−1^ and 23.3 min compared to 447 mAh g^−1^ and 40.6 min for PVA (Figure S12c,d).

Time and current are normalized by t_max_ and I_max_ based on the Scharifker‐Hills models in Figure 2j and compared with four classical electrochemical deposition models (Note S1) [71, 72]. The responses reveal that Li_2_S deposition on the PVA‐Cu cathode proceeds predominantly by a favourable 3D growth mechanism. This ensures the interface remains conductive and ion‐accessible, exposing sufficient catalytically‐active sites for the conversion of Li_2_S. In contrast, the PVA cathode exhibits a less favourable, mixed 2D/3D growth behaviour, more likely to form passivating films that limit kinetics and lead to incomplete conversions. This unfavourable deposition interface with slower kinetics aligns with the notably lower *D*_Li_^+^, the higher nucleation barrier and the prematurely terminated second discharge plateau for PVA. The accelerated conversion from LiPS to Li_2_S is crucial to maximise the sulfur utilization and further limits LiPS accumulation in the electrolyte, providing an additional mechanism to mitigate shuttle effects.

Overall, comparative electrochemical analyses demonstrate that PVA‐metal binders have a profound impact on reaction kinetics across all sulfur reduction and oxidation steps. Among the studied systems, PVA‐Cu consistently delivers the best overall performance, exhibiting the most efficient bidirectional catalytic activity for both reduction and oxidation reactions, while enabling greater capacity and faster Li_2_S deposition and dissolution.

### 2.3 High‐Rate and High‐Loading Li‐S Cells Enabled by Polymer‐Metal Complexes

At a practical sulfur loading of 3 mg cm^−2^ and rate of 1 C, the PVA‐Cu cell attains 1,119 mAh g^−1^ (3.4 mAh cm^−2^) with 82% capacity retention over 300 cycles (Figure 3a). In contrast, the PVA control cell exhibits a substantially lower capacity of 435 mAh g^−1^ after 300 cycles with only 55% capacity retention. In Figure 3b, at sulfur loadings of 1.5 mg cm^−2^, the PVA‐Cu cathode can operate at a high rate of 3 C, delivering an initial discharge capacity of 806 mAh g^−1^, retaining 62% of its capacity after 500 cycles with a Coulombic efficiency (CE) of >99.5%. Encouraged by its stable performance at moderate loadings, we further challenged the PVA‐Cu binder under higher sulfur areal loadings. At 4 mg cm^−2^, the cell delivers up to 780 mAh g^−1^ (3.1 mAh cm^−2^) over 200 cycles (Figure 3c), with excellent capacity retention of 95%. At an even more demanding loading of 6 mg cm^−2^, the cell delivers an initial areal capacity of 3.7 mAh cm^−2^ (622 mAh g^−1^) at 1 C—a demanding areal current density (10 mA cm^−2^) on both the cathode and the anode [73], retaining 70% of its capacity after 100 cycles (inset of Figure 3c).

**FIGURE 3:**
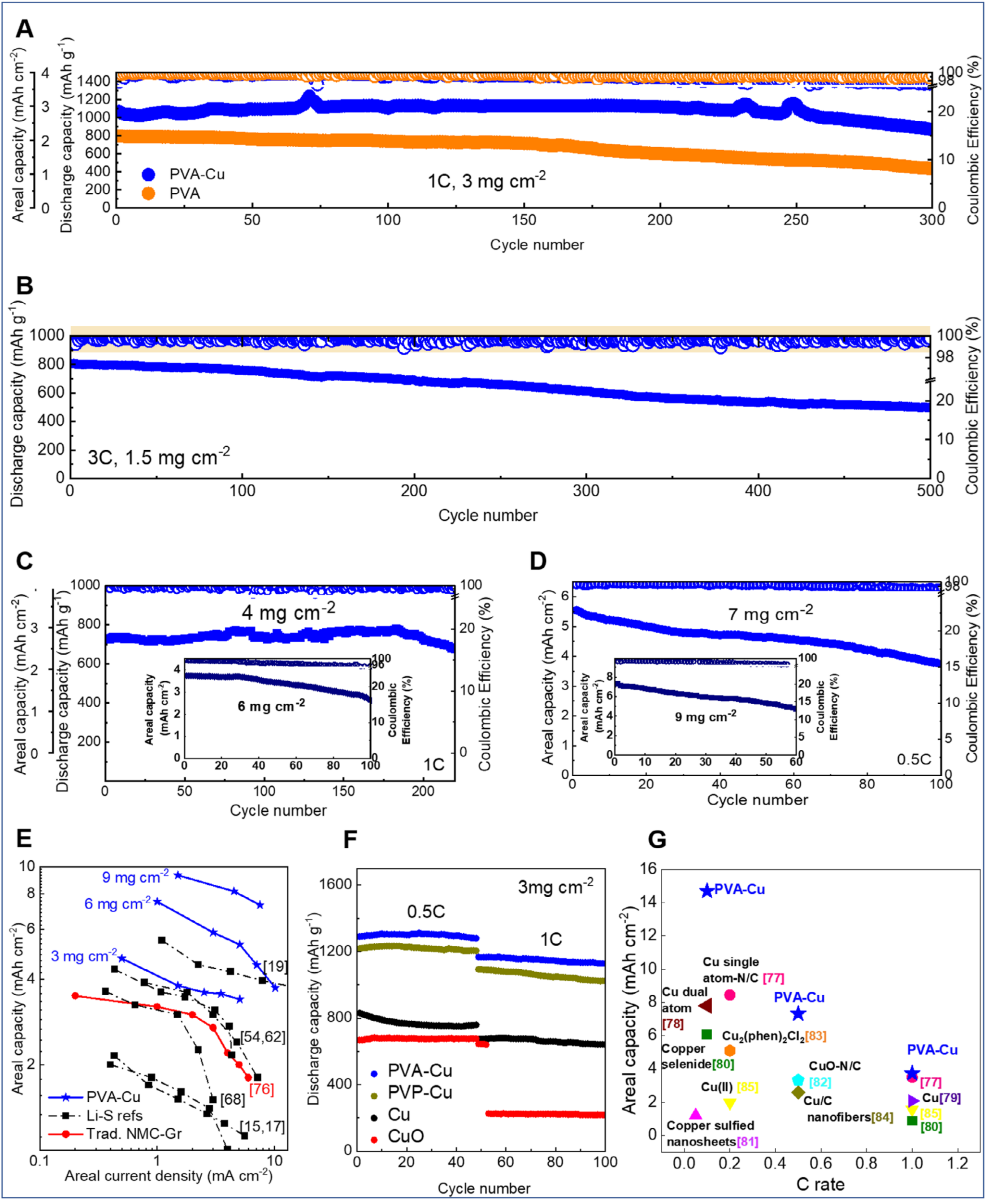
Performance of PVA‐Cu catalytic binder system at practical operating conditions. (A) 3 mg cm^−2^; (B) 1.5 mg cm^−2^; (C) 4 and 6 mg cm^−2^ sulfur loadings PVA‐Cu cathodes showing high discharge and areal capacities and extended cycle life at high current densities. (D) Further increase in sulfur loading to 7 and 9 mg cm^−2^ sulfur loading still deliver high areal capacities at 0.5 C rate. The cells in (A) and (B) were tested at an E/S ratio of ∼20 µL mg^−1^, those in (C) at 10–15 µL mg^−1^, and those in (D) at 7–8 µL mg^−1^. (E) Areal capacity achieved at increasing areal current densities demonstrates that the PVA‐Cu system is among the state‐of‐the‐art in the Li‐S literature, and even sit comparable to LIB benchmarks. (F) Cu‐polymer complexes (PVA‐Cu and PVP‐Cu) deliver higher discharge capacities than Cu based nanoparticles. (G) Comparison of the areal capacities of Cu‐polymer complexes with other state‐of‐the‐art Cu‐based heterogeneous and homogeneous catalysts across various current densities.

Post‐cycling SEM analysis of the cycled PVA‐Cu cathode in Figure S13 shows that the PVA‐Cu cathode maintains a well‐preserved morphology with no visible precipitation. In contrast, the control PVA cathode exhibits extensive surface deposition of inactive discharge products (dead sulfur). The accumulation of these insulating deposits impedes both ionic and electronic transport, resulting in the rapid capacity fading observed in the control sample [74]. Analysis of the lithium anode after cycling (Figure S14) provides inferential evidence of suppressed LiPS shuttling for the PVA‐Cu cell, which is clear from the smoother and more homogeneous surface morphology when compared to PVA, which exhibits significant corrosion and mossy dendritic growth. Moreover, EDX mapping of the PVA‐Cu lithium anode confirms the absence of Cu even after extended cycling (Figure S15), indicating that the polymer matrix effectively immobilizes Cu^2+^, even after extended cycling. These findings are further substantiated by X‐ray photoelectron spectroscopy (XPS) of the cycled lithium anodes (Table S4 and Figure S16). Namely, the PVA‐Cu anode exhibits a lower sulfur concentration (2.12 at%) than the PVA anode (9.35 at%), confirming reduced LiPS shuttling. In addition, the absence of a Cu signal further verifies that it remains immobilized within the polymer matrix throughout cycling.

At a loading of 7 mg cm^−2^, the cell delivers 791 mAh g^−1^ and 5.5 mAh cm^−2^ at 0.5 C for 100 stable cycles (Figure 3d). Challenging the PVA‐binder at 9 and 13 mg cm^−2^ loadings, areal capacities of 7.3 mAh cm^−2^ and 14.7 mAh cm^−2^ are achieved at 0.5 and 0.1 C, respectively (Figure 3d and Figure S17). These values meet or exceed the practical benchmark of ≥4 mAh cm^−2^ required for parity with commercial lithium‐ion battery technologies [75]. As summarized in Figure S18a, the areal capacity increases linearly with sulfur loading across the range of 2–9 mg cm^−2^ at 0.5 C, confirming efficient sulfur utilization and excellent electrode integrity even under thick‐electrode conditions, and a similar trend is observed at a lower rate of 0.1 C (Figure S18b).

Historically, Li‐S batteries have faced a persistent challenge in balancing the inherent trade‐off between rate capability, areal capacity and cycling stability. The PVA‐Cu system shows promise in overcoming this compromise and paves a step forward toward more practical sulfur cathodes. This is apparent when compared with previous reports in Figure S19. This advantage becomes clear when comparing the areal capacity and current density in Figure 3e, where the PVA‐Cu coin cells are among the state‐of‐the‐art in the Li‐S literature [15, 17, 19, 54, 62, 68], and established commercial LIB technologies such as NMC‐graphite [76].

To further elucidate and isolate the effect of the polymer coordination chemistry, we compared the PVA‐Cu binder with other Cu‐based analogues featuring similar metal sites, but fundamentally different surroundings: physically mixed Cu nanoparticles, CuO nanostructures without a polymer coordination environment and polyvinylpyrrolidone‐Cu (PVP‐Cu), another polymer‐metal complex. All samples contained equivalent Cu loadings (Table S5). In the polymer‐supported systems (PVA‐Cu and PVP‐Cu), the complexes are incorporated as part of the binder, whereas Cu and CuO nanoparticles are blended into the slurry.

At 0.5 C, the polymer‐supported catalysts (PVA‐Cu and PVP‐Cu) deliver substantially higher discharge capacities (>1,200 mAh g^−1^), compared with Cu and CuO, which delivered 830 and 682 mAh g^−1^, respectively (Figure 3f). At 1 C, the advantage is even more apparent. CV and GCD analyses of the *R*_1_, *R*_2_, and *O* processes show that PVA‐Cu exhibits higher peak currents and capacity contributions across all steps, indicating accelerated sulfur redox kinetics, consistent with its lower charge‐transfer resistance (*R*_ct_) (Figure S20a–e). These results confirm that polymer coordination of Cu effectively promotes both reduction and oxidation redox reactions in Li‐S electrochemistry, further highlighting the synergistic role of polymer‐metal coordination.

A broader comparison against Cu‐based catalysts reported in literature (Figure 3g) such as single‐atom [77] and dual‐ atom [78] catalysts, nano particles [79], selenides [80], sulfides [81], oxides [82] and others [83, 84, 85] shows that PVA‐Cu achieves among the highest areal capacities across all C‐rates (and further details are provided in Table S6). These results imply that, for a given Cu metal center, the PVA‐Cu system functions as a soft catalyst, simultaneously offering structural stability through a confined coordination network and a high density of accessible catalytic sites, enabling efficient sulfur redox kinetics for both high capacity and high‐rate operation.

### 2.4 Mechanistic Origins of Microenvironment‐Enhanced Catalysis

To unravel the mechanistic origins of the superior electrochemical behaviour of PVA‐Cu cathodes, we perform DFT calculations on the adsorption and electronic interaction of representative liquid‐ and solid‐phase Li‐S species with model PVA and PVA‐M (M = Cu, Zn, Fe, Ga, and Al) surfaces (Figures S21 and S22). The simulations included optimised atomic structures, binding energies, Bader charge analysis and electronic density of states (DOS) for PVA‐metal catalysts interacting with both liquid LiPS and solid lithium sulfides.

Single‐atom metal centres (Cu, Fe, Zn, Ga, and Al) anchored on the PVA‐derived polymer framework stabilise LiPS and Li_2_S primarily through adsorption by metal‐S and O‐Li hybridisation and charge transfer. Binding energies can quantify the strength of these interactions, and computed binding energies are summarised in Table S7. Our results show that all PVA‐metal binders exhibit more negative binding energies than PVA itself and bind exothermically, confirming that the PVA‐coordinated metal centre provides a polar microenvironment capable of immobilising LiPS and stabilising Li_2_S nuclei. However, the binding energies of these reactants exhibit different trends with various metal centres.

To bridge theory and experiment, we correlated the DFT‐calculated Li_2_S_6_ and Li_2_S binding energies for different PVA‐metal systems with the experimental Tafel slopes derived from the first and second cathodic peaks (*R*_1_ and *R*_2_) and the anodic peak (*O*). For peak *R*_1_ (Figure 4a), where Li_2_S_6_ is the dominant intermediate [74], the PVA‐Cu catalyst exhibits the lowest Tafel slope despite having a moderate binding energy of around 1.0 eV. In contrast, PVA‐Ga, Al and Zn exhibit much higher binding energies (>1.7 eV), indicating intrinsically stronger LiPS stabilisation, but have higher Tafel slopes. This is in line with the Sabatier principle, where optimal catalytic activity arises from intermediate binding strength [86, 87], such that the interaction is sufficient to weaken the intramolecular LiPS bonds to provide a lower‐energy reaction pathway but not too strong so as to facilitate product desorption and prevent surface passivation that might otherwise inhibit catalysis [3]. Although it should be noted that the Tafel slope and binding energies of Cu and Fe are not too dissimilar and reflect a similar first discharge plateau.

**FIGURE 4:**
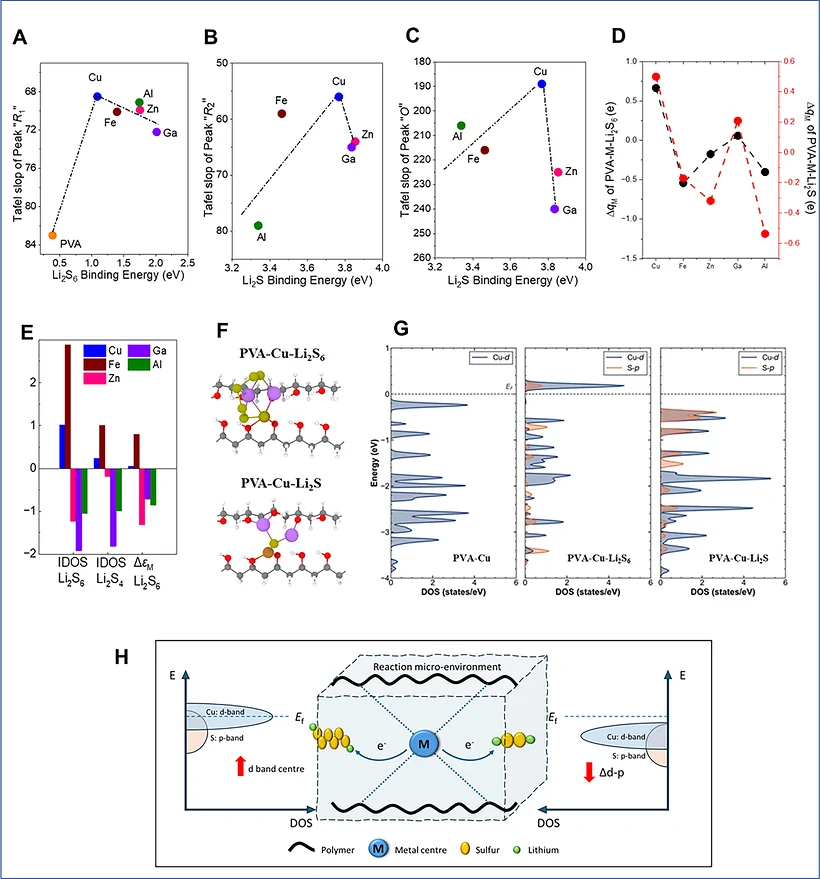
DFT calculations of the electronic structures of PVA‐metal catalysts with liquid and solid phase Li‐S reactants. Correlation between binding energies and Tafel slopes of PVA‐metal catalysts and PVA‐only system on Li_2_S_6_ and Li_2_S. (A) Tafel slope at first CV reduction peak vs. binding energies of Li_2_S_6_. (B) Tafel slope at second CV reduction peak vs. binding energies of Li_2_S and (C) Tafel slope at CV oxidation peak vs. binding energies of Li_2_S. (D) Adsorption‐induced Bader charge change (Δ*q*_M_) on the metal centre of PVA‐M‐Li_2_S_6_ and PVA‐M‐Li_2_S. (E) Integrated DOS (IDOS) and band centre shifts(Δ*ε*_M_) upon adsorption of Li_2_S_6_ and Li_2_S onto PVA‐M catalysts. (F) Optimised molecular configurations of PVA‐Cu‐Li_2_S_6_ and PVA‐Cu‐Li_2_S. (G) The projected density of states for Cu‐d orbitals with Li_2_S_6_ and Li_2_S. (H) Mechanism of polymer‐metal hybrids working as a catalytic binder system in Li‐S batteries, where catalytic properties of metal centres are combined with the structural advantages of the polymer matrix.

A similar trend is observed for Peak *R*_2_ and peak *O* (Figure 4b,c), where solid Li_2_S is the dominant species. However, the plot decreases more rapidly on either side of Cu, where for Fe and Al (lower Li_2_S binding than Cu), and for Ga and Zn (higher binding than Cu). Despite these variations, PVA‐Cu consistently exhibits the lowest Tafel slopes, in agreement with its distinctly longer second plateau. The moderate Li_2_S binding on PVA‐Cu facilitates a more favourable nucleation interface to guide a 3D distributed Li_2_S deposition morphology rather than dense, blocking films, but prevents extensive passivation of active sites by insulating Li_2_S layers. The dual‐site adsorption enabled by the PVA scaffold (O‐Li coordination for Li^+^ and Cu‐S chemisorption for S^2−^) together with the tailored electronic structure and enhanced charge transfer capability of the PVA‐Cu microenvironment, accelerates the Li_2_S formation and decomposition processes and supports highly reversible adsorption/desorption over extended cycling [88].

The better electrochemical performance of the PVA‐Cu is further supported by Bader charge analysis and the Fermi level shift of the d‐orbitals of single metal atoms upon adsorption of Li_2_S_6_ and Li_2_S. Charge transfer between adsorbates and active sites is a key determinant of Li‐S redox kinetics because it governs the degree of bond activation and the electronic accessibility of the surface. The metal‐centre response is highly metal‐dependent, and the net Bader charge at the metal centre (*q*_M_) and its adsorption‐induced change (Δ*q*_M_) relative to the clean PVA‐M slab are provided in Table S8. Cu exhibits the largest positive Δ*q*_M_ upon adsorption (Δ*q*_M_ = +1.500 e for Li_2_S and +0.663 e for Li_2_S_6_), indicating substantial oxidation of the Cu centre (Figure 4d). This signifies the highest degree of charge transfer from Cu to both liquid‐ and solid‐phase Li‐S species, consistent with optimal adsorption behaviour.

We further quantified the integrated DOS (IDOS) and the corresponding band‐centre shifts. Because Li‐S conversion reactions are multi‐electron processes, local electronic accessibility is as critical as chemical binding. Selected electronic‐structure descriptors extracted from DOS/projected density of states(PDOS analysis are given in Table S9. Notably, Li_2_S_6_ adsorption increases IDOS for Cu (from 1.00 to 2.02) and Fe (from 0.00 to 2.89), implying that LiPS coordination introduces additional states near the Fermi level and enhances local electronic delocalization (Figure 4e). Conversely, Li_2_S_6_ adsorption on Zn, Al and Ga reduces IDOS toward ∼1, suggesting partial electronic passivation even when adsorption is thermodynamically strong. The d band‐centre shifts support this distinction. Cu and Fe show an upward shift in *ε*_M_, whereas Zn and the p‐block metals show large downward shifts in *ε*_M_ upon Li_2_S_6_ adsorption (Figure 4e).

The optimised molecular configurations of PVA‐Cu systems with adsorbed Li_2_S_6_ and Li_2_S is illustrated in Figure 4f. As shown in Figure 4g, the PDOS reveals a pronounced overlap between the Cu d orbitals and the S p orbitals of Li_2_S_6._ This Cu‐S orbital hybridisation indicates stronger electronic coupling between the Cu metal centres and LiPS species, which stabilises the adsorbed LiPS and effectively suppresses the LiPS shuttle effect [89]. The increase in the d‐band centre, together with meta‐sulphur orbital hybridisation, provides an electronic basis for the experimentally observed enhancement in LiPS conversion kinetics, as the Cu d orbitals lower the energy barriers for LiPS conversion and thereby accelerate sulphur reduction reactions [90].

Li_2_S adsorbed on the PVA‐Cu system also exhibits clear metal‐sulfur orbital hybridisation, accompanied by a slight downward shift of the Cu d‐band centre (Figure 4g). Importantly, this shift is associated with a reduced energy gap between the Cu d orbitals and the Li_2_S p‐band centre. The narrowed energy gap facilitates enhanced interfacial electron‐transfer dynamics [90], which can be directly correlated with a reduced energy barrier for Li_2_S decomposition, thereby promoting oxidation during charging.

Our results reveal that polymer complexation is not a passive scaffold or structural feature, but an active determinant of redox dynamics by tuning the local polarity, orbital overlap, and charge transport, schematically depicted in Figure 4h. The PVA‐Cu microenvironment effectively modulates local DOS profiles, optimally balances d‐p orbital hybridisation, and narrows interfacial band gaps to lower kinetic barriers and improve long‐term cycling stability. These theoretical insights support the experimentally observed enhancement in electrochemical performance. The wider implication for sulfur cathodes is that deliberate design of a catalyst's molecular surrounding can transform an active centre into a more efficient catalyst. Looking forward, practical advances in sulfur electrocatalysis could rely on the modulation and design of the microenvironment to open a pathway toward simpler and more scalable, yet fundamentally effective catalyst architectures.

### 2.5 High‐Power Pouch Cell Prototypes

To validate the high‐rate performance of our catalytic PVA‐Cu binder system in a pouch cell and demonstrate its potential toward practical applications, we prepared three pouch cells and cycled at a 0.5 C rate. The detailed parameters of all pouch cells are summarised in Table S10. The first pouch cell is a 2‐layer, double‐sided cathode pouch cell prototype (Figure 5a) with a sulfur loading of 3.3 mg cm^−2^ on each side and an E/S ratio of 6. The cycling performance is shown in Figure 5b. The cells are initially cycled at 0.05 C, attaining 1,050 mAh g^−1^ and an energy density of 200 Wh kg^−1^, then at 0.1, 0.3, 0.4 C for a few cycles and 0.5 C for 20 cycles, delivering a discharge capacity of 790 mAh g^−1^ and recording an energy density of 140 Wh kg^−1^ at this rate. This is accompanied by a power density of 150 W kg^−1^. The energy densities were calculated by accounting for all active pouch‐cell components, excluding packaging materials and tabs, in accordance with standard literature practice. Power densities were determined using the actual discharge times of the cell. The GCD profiles maintain typical charge‐discharge curves with well‐developed profiles even at 0.5 C (Figure 5c). The second pouch cell, containing a total sulfur loading of 100 mg, delivered a maximum power density of 132 W kg^−1^ at 0.5 C and maintained 80% capacity retention over 100 cycles (Figure S23a,b). The third pouch cell, with a sulfur loading of 120 mg and a single electrode layer, also achieved a higher maximum power density of 140 W kg^−1^ at 0.5 C. This cell was cycled between 0.1 and 0.5 C and sustained stable operation for 60 cycles at 0.5 C (Figure S23c). Upon reducing the C‐rate back to 0.1 C, approximately 75% of the capacity was recovered. Given the challenges of running high‐rate Li‐S battery pouch cells, we compare the power density and C‐rate with those reported in the literature (Table S11). Figure 5d presents the power densities up to 0.5 C rate [54, 91, 92, 93, 94, 95, 96, 97, 98, 99, 100, 101, 102, 103, 104] and it is notable that although some reports achieve energy densities exceeding 300 Wh kg^−1^, their corresponding power densities remain below ∼10 W kg^−1^, reflecting the low cycling rates employed [91, 92, 93, 94].

**FIGURE 5:**
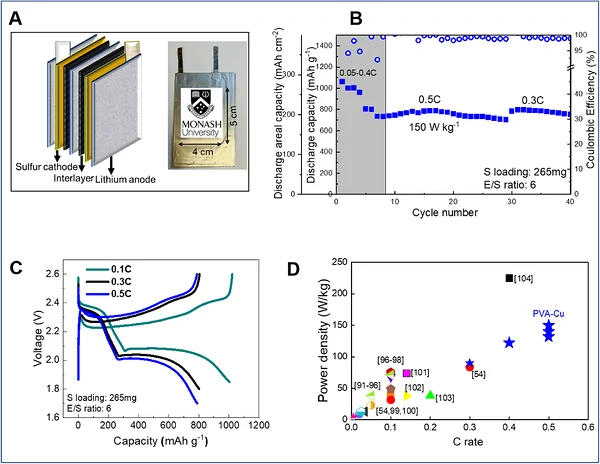
Power performance of Li‐S pouch cells employing a PVA‐Cu catalytic binder system. (A) Configuration and photo of double side coated two‐layer pouch cell. (B) Li‐S pouch cell containing a total sulfur loading of 265 mg, cycled at a rate of 0.5 C with an E/S ratio of 6. (C) Galvanostatic charge‐discharge profiles of the pouch cell recorded at 0.1, 0.3, and 0.5 C rates. (D) Comparison of the power density achieved with the PVA‐Cu‐based cathode relative to previously reported Li‐S pouch cells.

## 3 Conclusions

This work establishes polymer‐metal coordination catalysis as a generic design principle for efficient bidirectional sulfur redox chemistry. While subtle, this strategy effectively modulates the reaction microenvironment. We demonstrate that soft polymer‐metal catalysts provide a practical and versatile strategy for high‐performance Li‐S batteries under realistic operating conditions. Our model system, combining PVA with coordinated metal centres, effectively addresses the intrinsically sluggish reduction and oxidation kinetics, enabling high‐rate operation at higher loading. We identify that PVA‐Cu is notably efficient at accelerating both the reduction and oxidation redox kinetics, and report among the highest areal capacities at 0.5 C (delivering 7.3 mAh cm^−2^ at 9 mg cm^−2^) and 1 C (delivering 3.7 mAh cm^−2^ at 6 mg cm^−2^) in that system. This performance is attributed to the synergistic interplay of factors at the atomic and microstructural levels. The polar microenvironment of PVA‐Cu offers a three‐dimensional binding environment through cooperative O‐Li coordination and Cu‐S chemisorption, while simultaneously introducing additional states near the Fermi level. This enables efficient electron and ion transport and sustained catalytic activity, delivering enhanced sulfur utilisation throughout cycling even at higher rates. We demonstrate that the PVA‐Cu binder is directly translatable to pouch cell devices, enabling stable cycling at 0.5 C. To the best of our knowledge, the resulting performance are in the upper range of contemporary literature benchmarks for high‐rate Li‐S prototype devices, placing this device among the highest‐rate cells reported to date. Overall, the concept of soft catalysis via polymer‐metal coordination offers a promising design paradigm, opening new directions for future research in advanced Li‐S battery technologies.

## 4 Experimental Section

### 4.1 Materials

The cathode was comprised of elemental sulfur (Sigma–Aldrich), carbon ((CABOT black pearl 2000, Shandong Gelon LIB Co., LTD, China), Acetylene‐black, and kurrary carbon) and binder composed of polymer Poly(vinyl alcohol) (PVA) polymer; average M_w_ 89,000‐98,000 (Sigma–Aldrich), one of the metal nitrates; (Copper(II) nitrate trihydrate, Zinc nitrate hexahydrate, Iron(III) nitrate nonahydrate, Aluminum nitrate nonahydrate, Gallium(III) nitrate hydrate (Sigma–Aldrich), and Carboxymethylcellulose (CMC) (Sigma–Aldrich). Further variant was prepared by replacing PVA with Polyvinylpyrrolidone (PVP)polymer; average M_w_ 360 000 (Sigma–Aldrich) with Copper(II) nitrate trihydrate. Even further variants were prepared by using Copper nanopowder, 25 nm (Sigma–Aldrich), and Copper oxide nanopowder, 50 nm (Sigma–Aldrich) as additives, and CMC as a binder system. Carbon‐coated glass fibre interlayer comprised of carbon (ASAC30, Adven Industries Inc., Canada), Gum Arabic (HawkinsWatts) and glass fibre (BG03013 separator, Hollingsworth & Vose, USA). Bis (trifluoromethane) sulphonamide lithium salt and lithium nitrate were purchased from Sigma–Aldrich and directly used without any further purification. DME and DOL solvent was purchased from Sigma–Aldrich. Li_2_S was purchased from Alfa Aesar for lithium polysulfide synthesis. Battery‐grade Al foil was purchased from Japan Capacitor Industrial Co., Celgard 2730 separator was purchased from Celgard Inc., USA. Lithium chips (16*0.2 mm) was purchased from Shandong Gelon LIB Co., LTD, China.

### 4.2 Preparation of Polymer‐Metal Hybrid Binder Systems

To synthesise the PVA‐metal complexes, 4.5 wt.% poly(vinyl alcohol) (PVA) was dissolved in 2 mL of deionised water by stirring at 80°C–90°C for 1 h using a magnetic stirrer. After complete dissolution, the solution was cooled to room temperature before adding the appropriate metal nitrate to form the PVA‐metal complex. For the PVA‐Cu system, 6.4 wt.% of Cu(NO_3_)_2_.3H_2_O (4.7% wt Cu(NO_3_)_2,_ equivalent to 0.25 mol of Cu^2+^) was added and stirred for 2 h. The same procedure was followed to prepare PVA‐metal complexes using Zn(NO_3_)_2_.6H_2_O (7.4 wt.%), Fe(NO_3_)_3_.9H_2_O (10.1 wt.%), Ga(NO_3_)_3_.xH_2_O (6.4 wt.%), and Al(NO_3_)_3_.9H_2_O (9.4 wt.%), each added in an amount corresponding to 0.25 mol of metal ions. All solutions were stirred for 2 h after metal nitrate addition to ensure the formation of stable PVA‐metal complexes.

### 4.3 Characterization of Polymer‐Metal Complexes

Experimental and theoretical studies were conducted to confirm the complexation between PVA and various metal cations. Each PVA‐metal complex solution was drop‐cast onto glass slides, dried at room temperature, and analysed using Fourier Transform Infrared Spectroscopy (FTIR) and X‐ray Diffraction (XRD). FTIR spectra were recorded using an attenuated total reflectance (ATR) FTIR spectrometer (PerkinElmer, USA) in the range of 400–4000 cm^−1^ with an average of 32 scans per sample. XRD patterns were obtained using a Bruker D8 Advance Diffractometer XRD under Cu Kα radiation generated at 40 kV and 10 mA with a scan rate of 0.2 min^−1^ and a step size of 0.01 to investigate the structural changes. The molecular structure of a PVA‐metal complex was further investigated via reactive molecular dynamics simulations using the ReaxFF force field [105]. A bilayer PVA model was constructed and optimised through molecular dynamics and conjugate gradient energy minimisation on Amsterdam Modelling Suite. To simulate the PVA‐Cu complex, two protons were removed from the optimised bilayer PVA and a Cu^2+^ ion was placed approximately 5 Å above the deprotonated site to form a charge‐neutral assembly. An additional proton was removed for PVA‐metal complexes involving trivalent metal ions (Al^3+^, Ga^3+^, and Fe^3+^), and the Cu^2+^ ion was replaced with the respective trivalent metal ion to maintain overall charge neutrality.

### 4.4 Preparation of Sulfur Cathodes With PVA‐Metal Catalytic Binders

A mixture of sulfur (65 wt.%), conductive carbon (20 wt.%), and an appropriate amount of CMC was stirred for 24 h using a magnetic stirrer to ensure uniform dispersion. After this initial mixing, 2 mL of each PVA‐metal binder solution was added to the dry mixture, and the slurry was stirred for an additional 24 h at 600 rpm under ambient temperature and dry conditions to obtain a homogeneous slurry. Each binder system comprising the PVA‐metal complex and CMC, constituted 15 wt.% of the total slurry. (Each binder system in 2 mL of DI water was used for 1 g of dry components in the final slurry for all slurries.) The resulting slurries were then ready for electrode fabrication. The slurries were coated onto battery‐grade aluminum foil using the doctor blade technique and dried at room temperature for approximately 10 h, followed by vacuum drying at 70°C for 12 h. The sulfur loading of the prepared cathodes (1 cm x 1 cm, coin cell configuration) ranged from 3 to 13 mg cm^−2^. Six different types of sulphur cathodes, including control samples without metal additives and samples containing different PVA‐metal binders, are summarised in Table S1. The cathode formulation was kept consistent across all electrochemical tests for all cathode systems.

### 4.5 Preparation of Sulfur Cathodes With Different Cu‐Based Systems

Sulfur cathodes incorporating various Cu‐based systems were prepared following the same procedure used for PVA‐metal cathodes. For PVP‐Cu cathodes, PVA was replaced with polyvinylpyrrolidone (PVP), and the synthesis followed the same steps as for the PVA‐Cu binder system. Additional cathodes containing Cu and CuO nanostructures were fabricated without using any polymeric metal‐binding agents (PVA or PVP) and employed CMC as the binder system. In these formulations, the total Cu content was kept at 1.6 wt.% relative to the dry components, matching the Cu content in both the PVA‐Cu and PVP‐Cu cathodes, to ensure consistency across all samples. A detailed summary of all cathode compositions involving different Cu‐based systems is provided in Table S4.

### 4.6 Coin Cell Assembly

Coin cells were assembled in an argon‐filled glovebox with O_2_ and moisture <0.1 ppm. Lithium metal was employed as the counter electrode, and a Celgard separator. A glass fiber interlayer was used, which had been coated with an aqueous slurry composed of activated carbon (80 wt.%) and Gum Arabic (20 wt.%), followed by vacuum drying at 100°C overnight to eliminate residual moisture. The carbon loading on the interlayer ranged between 1.0 and 1.5 mg cm^−2^. The electrolyte consisted of 0.4 m lithium bis(trifluoromethanesulfonyl)imide (LiTFSI) and 0.6 m lithium nitrate (LiNO_3_) dissolved in a 1:1 volume ratio of 1,3‐dioxolane (DOL) and dimethoxyethane (DME). The E/S ratio was adjusted between 5 and 20 µL mg^−1^ depending on the sulfur loading of each cathode. For example, low sulfur‐loading cathodes (1–3 mg cm^−2^) were tested at an E/S ratio of approximately 20 µL mg^−1^, intermediate loadings (4–6 mg cm^−2^) between 10–15 µL mg^−1^, and higher loadings (7–13 mg cm^−2^) at around 5–8 µL mg^−1^.

### 4.7 Pouch Cell Assembly

The cathode slurry was coated on both sides of Al foil to prepare double‐sided sulfur cathodes. These double‐sided electrodes were used for pouch‐cell fabrication, and an Al tab was welded onto the sulfur cathode. A carbon‐coated thin glass‐fiber interlayer was then attached to the double‐sided cathode, followed by stacking with a Celgard separator. The assembled stacks were transferred into an Ar‐filled glove box, where Li‐metal anodes were placed on the opposite side of the Celgard separator. Lithium foil with a thickness of 0.1 mm was used as the anode, and a Ni tab was bonded to the Li anode using a two‐spot welder. The electrolyte was injected into the cell stack, and the pouch cells were sealed under vacuum inside the glove box. All pouch cells were assembled in an Ar‐containing glove box (<0.1 ppm H_2_O and <0.1 ppm O_2_). All the parameters of pouch cells are given in Table S3.

The gravimetric energy density (specific energy) of the pouch cells was calculated using the formula *E*_g_ = *VC*/(∑mi), where *E*_g_ is the specific energy (Wh kg^−1^), *V* is the output voltage (V), *C* is the output capacity (mAh) and ∑mi is the total weight of the pouch (cathode, anode, interlayer, separator and electrolyte. The power density/specific power (W kg^−1^) was then calculated by dividing the corresponding energy density by the actual discharge time (h) of each pouch cell.

### 4.8 Electrochemical Characterization

A multichannel battery testing system (Neware, China) was used to get the galvanostatic charge‐discharge data between 1.8–2.8 V. Cyclic voltammetry (CV) was carried out between scan rate of 0.05 to 0.1 mV s^−1^ from 1.8 to 2.8 V (vs. Li^+^/Li) at room temperature. The Lithium ion diffusion coefficient (𝐷_Li+_ in cm^2^ s^−1^) was calculated according to the Randles‐Sevick equation, where the power‐law relationship between the peak current obtained from CV measurements and the square root of the scan rate: 𝐼p = 2.69 × 10^5^𝑛^1.5^𝐴$D_{Li+}^{0.5}$ 𝐶_Li_𝑣^0.5^. 𝐼p is the peak current(A), n is the number of electrons in the redox reaction (n = 2 in Li‐S batteries), A is the electrode area (1 cm^2^ in this work), 𝐶_Li_ is the Lithium‐ion concentration in the electrolyte (mol mL^−1^) and V is the Scanning rate (V s^−1^). Electrochemical impedance spectroscopy (EIS) tests were carried out on EC‐Lab, measuring responses over frequencies between 1 and 10 mHz, recording six points per decade of frequency, 10 mV rms alternating currents (AC) voltage and 2.8 V vs E_ref_ direct current (DC) voltage. Temperature dependence of *R*_ct_ at potentials between 2.8 and 1.8 V during battery discharge is fitted according to the Arrhenius equation to calculate the activation energies.

### 4.9 Synthesis of Li_2_S_6_ and Li_2_S_8_ Solutions

The synthesis of Li_2_S_6_ and Li_2_S_8_ solutions was followed from the method reported by Liao et al. [106] and Sun et al. [107], respectively. The Li_2_S_6_ solution was prepared by dissolving elemental sulfur and lithium sulfide (Li_2_S) in a 1:1 (v/v) mixture of DOL and DME. The reactants were combined in a molar ratio of 8:5 (8 Li_2_S + 5S_8_ → 8 Li_2_S_6_) and stirred continuously at 50°C for 36 h inside an argon‐filled glovebox. After reaction completion, the mixture was centrifuged at 5,000 rpm for 10 min to remove any undissolved particles, and the resulting red‐brown supernatant was collected as the Li_2_S_6_ solution. To prepare the Li_2_S_8_ solution, 0.92 g of Li_2_S and 4.48 g of sulfur were dissolved in DOL/DME (1:1 v/v) under continuous stirring at 55 °C for 48 h, yielding a 0.5 m Li_2_S_8_ solution.

### 4.10 Li_2_S_6_ Symmetric Cell Assembly and Measurements

Symmetrical cells were assembled without sulfur in the electrodes. Conductive carbon and each PVA‐metal binder system (or the control PVA binder) were mixed in a 3:1 dry weight ratio to prepare electrode slurries. These slurries were then coated and dried using the same method applied for sulfur cathodes. Two identical electrodes (1 cm x 1 cm) were used as both the working and counter electrodes. A Celgard membrane was used as the separator. Each electrode was wetted with 10 µL of 0.5 m Li_2_S_6_ and 15 µL of the conventional electrolyte used in Li‐S coin cells. CV measurements were performed at a scan rate of 3 mV s^−1^ over a voltage window from −0.8 to 0.8 V. EIS measurements for the symmetric cells were performed using EC‐Lab, under the same parameters as those used for the sulfur cathodes.

### 4.11 Li_2_S_8_ Cell Assembly and Li_2_S Nucleation and Dissolution Assessment

To assemble the cells, electrodes prepared with PVA‐Cu catalyst binder and only PVA binder systems were used in symmetric cell experiments (1 cm x 1 cm). A lithium foil served as the counter electrode, and a Celgard membrane functioned as the separator. 15 µL of Li_2_S_8_ solution (0.5 mol L^−1^) was used as the catholyte. Anolyte consisted of the conventional electrolyte used in Li‐S coin cells. For Li_2_S tests, the cells were galvanostatically discharged to 2.09 V and subsequently potentiostatically discharged at 2.08 V until the current dropped to 10^−5^ A. For Li_2_S dissolution tests, the cells were first galvanostatically discharged to 1.8 V, then charged potentiostatically at 2.4 V until the current was below 10^−5^ A.

D_Li_^+^ values were calculated in GIIT by using the formula D_GITT_ = (4/πt) x (mB/ρS)^2^ x (ΔEs/ΔEt)^2^, where ρ is the density of the sulfur (2.07 g/cm^3^), mB is the mass of active material in the electrode (0.004 g), and S is the contact area between the electrode and the electrolyte (1 cm^2^).

### 4.12 Scanning Electron Microscopy Imaging and EDX Mapping

Secondary electron imaging and energy‐dispersive x‐ray spectroscopy (EDX) mapping of fresh sulfur cathodes were performed using a Thermo Scientific Verios 5 UC FEGSEM. Post‐cycling lithium anodes were imaged using a Nova 450 field emission scanning electron microscope (FESEM).

### 4.13 Conductivity Measurements

The sheet resistance of cathodes with the same coating thickness was measured using Everbeing SR 4L four‐point prober, Keithley four‐point probe and Keithley 237 source measure unit under a source current of 1 mA.

### 4.14 First Principal Calculations

First‐principles calculations were performed using the Vienna Ab initio Simulation Package (VASP) within the projector‐augmented wave (PAW) formalism. Exchange‐correlation interactions were treated within the generalized‐gradient approximation using the Perdew‐Burke‐Ernzerhof (PBE) functional. A plane‐wave kinetic energy cutoff of 520 eV and accurate Fourier grids were used throughout. Spin polarization was enabled, with initial magnetic moments assigned to facilitate self‐consistent convergence for open‐shell metal centres. Non‐spherical gradient corrections inside the PAW spheres were included and symmetry was disabled to avoid artefacts in low‐symmetry polymer‐metal slab models. Periodic slab models of PVA‐coordinated single metal sites (Cu, Zn, Al, Ga, and Fe) with adsorbed Li‐S species were constructed in an orthorhombic supercell with the surface normal along z. A 50 Å cell dimension along z was employed to ensure a sufficiently thick vacuum region for surface electrostatics. All structures were relaxed using the conjugate‐gradient algorithm and electronic and ionic convergence criteria were set to 1×10^−5^ eV and 0.02 eV Å^−1,^ respectively. Brillouin‐zone integrations for geometry optimization used a Gamma‐centered Monkhorst‐Pack mesh of 4×1×1. Gaussian smearing was applied. Projected densities of states were obtained with Gaussian smearing with 0.05 eV width and a Gamma‐centered Monkhorst‐Pack mesh of 20×1×1. Charge transfer between the PVA‐metal sites and Li‐S adsorbates was quantified using Bader charge partitioning. Methfessel‐Paxton smearing was employed for the Bader single‐point runs. Bader charges were computed using the grid‐based algorithm of Henkelman and co‐workers, and net atomic charges were reported as *q_i_ = ZVAL_i_—N_i_* (Bader). Adsorption thermodynamics were quantified using binding energies computed as *E_b_ = E_(slab + X)_—E_(slab)_—E_(Xgas)_*, where *X* denotes Li_2_S or Li_2_S_6_. Negative binding energies indicate exothermic adsorption and stronger interfacial stabilization.

## Author Contributions

M.M.N., P.J., M.S., and M.M. conceived the project. M.M.N., P.J., M.S., M.H. and M.M. designed the experiments. M.M.N., P.J., and D.M. ran the experiments. S.D. and A.T. built structural models, performed DFT‐based calculations, and analysed their results. M.M.N., P.J., M.S., D.M., M.H. and M.M. analysed the data. M.M.N., P.J., M.S., and M.M. wrote the manuscript. P.J., M.S., and M.M. oversaw the project.

## Conflicts of Interest

M.M.N., P.J., M.S., and M.M. are the authors of a patent application (PCT/AU2024/051384). The other authors declare no competing interests.

## Supporting information




**Supporting File**: advs77302‐sup‐0001‐SuppMat.docx.

## Data Availability

The data that support the findings of this study are available on request from the corresponding author. The data are not publicly available due to privacy or ethical restrictions.
