# Supplementary material for: Bidirectional Soft Catalysts for Improved Power Performance of Li‐S Batteries
Source: Adv Sci (Weinh). 2026 Aug 21;13(62):e77302. doi: 10.1002/advs.77302 (PMC13496154; doi:10.1002/advs.77302)
Supplement: Supplementary file 1 [file ADVS-13-e77302-s001.docx]

**Bidirectional soft catalysts for improved power performance of Li-S batteries**

*Maleesha M. Nishshanke^1^, Petar Jovanovic^1,2*^, Swarit Dwivedi^3,4^, Declan McNamara^3^,* *Akshat Tanksale^4^, Mahdokht Shaibani^1,2,5^, Matthew Hill^3^, Mainak Majumder^1,2,*^*

*^1^Nanoscale Science and Engineering Laboratory (NSEL), Department of Mechanical and Aerospace Engineering, Monash University, Clayton, VIC 3800, Australia*

*^2^ARC Research Hub for Advanced Manufacturing with 2D Materials, Monash University, 20 Research Way, Clayton, VIC 3800, Australia*

*^3^Department of Materials Science and Engineering, Monash University, Clayton, VIC 3800, Australia*

*^4^Department of Chemical & Biological Engineering, Monash University, Clayton, VIC 3800, Australia*

*^5^Department of Chemical and Environmental Engineering, RMIT University, Melbourne, VIC 3001 Australia*

*^*^Email:*[*mainak.majumder@monash.edu*](mailto:mainak.majumder@monash.edu)*,* [*petar.jovanovic@monash.edu*](mailto:petar.jovanovic@monash.edu)

**Supporting Tables**

Table S1. Description of all cathodes with different PVA-metal and control binder compositions

| **Cathode** | **Cathode compositions with proportions (%)** | | | | |
| --- | --- | --- | --- | --- | --- |
|  | **Sulfur** | **Carbon** | **Binder** | | |
|  |  |  | **PVA** | **Each Metal nitrate (excluding .xH_2_O)** | **CMC** |
| PVA-Cu | 65.00 | 20.00 | 4.50 | 4.69 | 5.81 |
| PVA-Fe | 65.00 | 20.00 | 4.50 | 6.05 | 4.45 |
| PVA-Zn | 65.00 | 20.00 | 4.50 | 4.73 | 5.77 |
| PVA-Ga | 65.00 | 20.00 | 4.50 | 6.39 | 4.11 |
| PVA-Al | 65.00 | 20.00 | 4.50 | 5.32 | 5.18 |
| PVA | 65.00 | 20.00 | 4.50 | - | 10.50 |

Table S2. Electrode properties of PVA and PVA-Cu. Values represent the average of three independent measurements.

| Electrode | *ρ* (g cm^-3^) | *ε (%)* | *τ* | *R_ion_* *Ω cm^2^* |
| --- | --- | --- | --- | --- |
| PVA-Cu | 0.74±0.016 | 61.7±0.8 | 4.6±0.4 | 8.2±0.6 |
| PVA | 0.68±0.007 | 63.8±0.4 | 6.8±1.3 | 11.9±2.2 |

To investigate the influence of polymer-metal coordination on electrode porosity, tortuosity and ion transport, electrochemical impedance spectroscopy (EIS) measurements were performed to quantify the ionic resistance within the porous electrode (*R*_ion_) and the corresponding tortuosity (*τ*). The electrode porosity (*ε*) was determined from the relationship between the measured bulk density of the coated electrode (*ρ*_bulk_), including pore volume, and the theoretical density of the solid electrode components (*ρ*_theoretical_), according to *ε* = 1 - *ρ*_bulk_/ρ_theoretical_ [1].

To eliminate the overlapping impedance contributions from the lithium anode and sulfur cathode in full cells, symmetric cathode|cathode cells were employed for the measurements. The ionic resistance within the porous electrode was obtained from the real-axis projection of the impedance response, where *R*_ion_/3 corresponds to the ion-transport resistance in the electrode pores [2]. The tortuosity factor was calculated using *τ* = (*R*_ion_A*εκ*)/2L, where A is the electrode area, *κ* is the electrolyte ionic conductivity and L is the electrode thickness [3].

Table S3: Sheet resistance of electrodes.

| Cathode | Sheet resistance (Ω sq^-1^) |
| --- | --- |
| PVA | 95.16±9.8 |
| PVA-Cu | 10.57±1.5 |
| PVA-Fe | 12.38±1.2 |
| PVA-Zn | 29.98±1.4 |
| PVA-Ga | 24.85±3.2 |
| PVA-Al | 15.16±1.8 |

Table S4: The atomic percentages of S2p, O1s, F1s, C1s, and N1s on the cycled Li metal anode.

|  | Atomic % | | | | |
| --- | --- | --- | --- | --- | --- |
|  | S2p | O1s | F1s | C1s | N1s |
| PVA | 9.35 | 37.04 | 7.86 | 41.86 | 3.89 |
| PVA-Cu | 2.12 | 27.68 | 6.62 | 33.24 | 1.8 |

Table S5. Description of all cathodes with different Cu-based compositions.

| **Cathode** | **Cathode compositions with proportions (%)** | | | | |
| --- | --- | --- | --- | --- | --- |
|  | **Sulfur** | **Carbon** | **Binder** | | |
|  |  |  | **Metal-binding polymer** | **Cu** | **CMC** |
| PVA-Cu | 65.0 | 20.0 | PVA-4.5 | Cu(NO₃)₂·3H₂O=6.4  (Cu(NO₃)₂ = 4.7) | 5.8 |
| PVP-Cu | 65.0 | 20.0 | PVP-4.5 | Cu(NO₃)₂·3H₂O=6.4  (Cu(NO₃)₂ = 4.7) | 5.8 |
| Cu | 65.0 | 20.0 | - | Cu=1.6 | 13.4 |
| CuO | 65.0 | 20.0 | - | CuO=2.0 | 13.0 |

Table S6. Comparison of the cell performance with other recently reported Cu-based Li-S studies.

| Study | S loading  (mg cm^-2^) | rate | cycle | E/S  μL mg^-1^ | Areal capacity  (mAh cm^−2^) | Discharge capacity | Reference |
| --- | --- | --- | --- | --- | --- | --- | --- |
| Copper dual-atom catalyst | 5.1 | 0.1 | 50 | - | 5.1 | - | Nature Communications-2024, 15:3231 |
|  | 7.7 | 0.1 | 50 | 4.8 | 7.8 | 1012.98 |  |
|  | 1.4-1.6 | 2 | 400 | 15 | 1.36 | 1140.6 |  |
| Copper‐carbon nanofibers interlayer | 1.5-2 | 0.5 | 300 | 15 | ~2.6 | 1300 to 626 | Carbon Energy-2024, 6: 450. |
|  | 3.8 | 0.1 | 100 | 10 | 4.25 | 1144 |  |
| Cu-ETL-INT | N/A | 3 | 500 | 12 | N/A | 600 | Angew. Chem-2024, e202415078 (2 of 11) |
|  | 7.8 | 0.2 | 100 | 4.8 | 7 | ~897.43 |  |
| Cu-MOFs | N/A | 0.5 | 200 | - |  | 954-250 | Journal of Electroanalytical Chemistry-2024, 967, 118468 |
| (Cu2(phen)2Cl2) | 6.5 | 0.2 | 60 | 4.5 | 5.1-4.1 | ~600 | Advanced Materials -  2024, 36, 2405790 |
| CuO@NC | 3.43 | 0.5 | 200 | 26 | 3.33 | 969.8 | Electrochimica Acta-  2025, 521: 145936 |
| C/Cu@S | 2.0 | 1 | 400 | 20 | 2.08 | 1039 | Electrochimica Acta -  2024, 507:145099 |
| S/Cu_1.8_Se | 7.2 | 0.1 | 100 | - | 6.07 | 843.05 | ACS Nano-2022,  16 (7), 11102–11114 |
| Macrocyclic Cu(II) complex | 10.4 |  | 70 | 6 | 9.5 | ~925 | Advanced Materials -2023, 35 (26): 2300861 |
|  | 2 | 1 | 200 | - | 1.56 | 780-601 |  |
| Copper sulfide nanosheets | 1 | 0.05 |  |  | 1.2 | 1205 | Mater. Chem. A.-  2022, 10,4015-4023 |
|  | 5 | - | 100 | 7.5 | 5.7 | 1130 |  |
| SA-Cu @ N-doped carbon | 4 | 5 | 500 | 10 | ~2.4-1.6 | ~650 | Energy Storage Materials -2022, 51, 890-899 |
|  | 10 | 0.2 | 100 | 6 | 8.44 | 844 |  |
| PVA-Cu | 3 | 1 | 300 | 20 | 3.4 | 1133 | Our work |
|  | 6 | 1 | 100 | 10 | 3.7 | 621.8 |  |
|  | 4 | 1 | 200 | 15 | 3.1 | 780 |  |
|  | 7 | 0.5 | 100 | 8 | 5.5 | 791 |  |
|  | 9 | 0.5 | 60 | 7 | 7.3 | 813 |  |
|  | 13 | 0.1 | 40 | 5 | 14.7 | 1101 |  |

Table S7. Binding energies (*E_b_*) of Li_2_S and Li_2_S_6_ on PVA-M surfaces.

| Metal (M) | *E_b_* (Li_2_S) (eV) | *E_b_* (Li_2_S_6_) (eV) |
| --- | --- | --- |
| Cu | -3.767 | -1.088 |
| Zn | -3.854 | -1.754 |
| Al | -3.339 | -1.744 |
| Ga | -3.835 | -2.014 |
| Fe | -3.464 | -1.395 |
| PVA | -1.720 | -0.390 |

Table S8. Metal-centre Bader charges and adsorption-induced charge shifts.

| Metal | *q_M_*(slab) (e) | *q_M_* (Li_2_S) (e) | *Δq_M_* (Li_2_S) (e) | *q_M_* (Li_2_S_6_) (e) | *Δq_M_* (Li_2_S_6_) (e) |
| --- | --- | --- | --- | --- | --- |
| Cu | 1.129 | 1.629 | 0.500 | 1.791 | 0.663 |
| Zn | 1.196 | 0.877 | -0.319 | 1.021 | -0.175 |
| Al | 2.516 | 1.980 | -0.536 | 2.114 | -0.402 |
| Ga | 1.164 | 1.373 | 0.209 | 1.221 | 0.058 |
| Fe | 1.517 | 1.347 | -0.171 | 0.975 | -0.542 |

Table S9. Selected electronic-structure descriptors extracted from DOS/PDOS analysis.

| Metal | I_DOS_ (slab) | I_DOS_ (Li_2_S) | I_DOS_ (Li_2_S_6_) | *εM*(slab) (eV) | *εM* (Li_2_S_6_) (eV) | *ΔεM* (Li_2_S_6_) (eV) |
| --- | --- | --- | --- | --- | --- | --- |
| Cu | 1.000 | 1.232 | 2.019 | -2.404 | -2.355 | 0.049 |
| Zn | 2.234 | 2.049 | 1.001 | -4.911 | -6.230 | -1.319 |
| Al | 2.056 | 1.061 | 1.001 | -6.109 | -6.963 | -0.854 |
| Ga | 2.917 | 1.101 | 0.999 | -5.615 | -6.330 | -0.715 |
| Fe | 0.000 | 0.999 | 2.890 | -3.273 | -2.479 | 0.794 |

Table S10. The parameters of 0.5 C pouch cells.

| Parameters | 0.5C Pouch-1 | 0.5C Pouch-2 | 0.5C Pouch-3 | Description |
| --- | --- | --- | --- | --- |
| Sulfur amount | 103 | 120 | 265 | mg |
| Cathode size | 5×3 | 5×3 | 5×4 | cm × cm |
| Layers of cathode | 1 | 1 | 2 | Double-sided |
| Areal sulfur loading  (Double side) | 6.8 | 8.0 | 6.6 | mg/cm^2^ |
| Weight of cathode (with Al current collector) | 257 | 280 | 669 | mg |
| Anode size | 5×3.3 | 5×3.3 | 5×4.3 | cm × cm |
| Li thickness | 100 | 100 | 100 | µm |
| Layers of anode | 2 | 2 | 3 |  |
| Weight of anode | 130 | 120 | 255 | mg |
| N/P ratio | 3.9 | 3.4 | 3.0 |  |
| Weight of separator | 140 | 130 | 246 | Celgard 2730 |
| Weight of Interlayer | 110 | 140 | 250 | mg |
| E/S ratio | 7 | 7 | 6 |  |
| Nominal voltage | 2.04 | 2.08 | 2.01 | V |
| Weight of electrolyte | 700 | 840 | 1590 | mg |
| Total weight (cathode+ anode+ separator+  interlayer+ electrolyte) | 1337 | 1510 | 3010 | mg |
| Peak-specific capacity | 549.7 | 632.2 | 789.3 | mAh/g |
| Capacity | 57.0 | 76.0 | 209.2 | mAh |
| Areal Capacity (double side) | 3.7 | 5.0 | 5.2 | mAh/cm^2^ |
| Specific energy | 86.0 | 105.0 | 140.0 | Wh/Kg |
| Specific power | 132 | 140 | 150.0 | W/Kg |

Table S11: Energy density and power density performance of reported Li-S pouch cells.

| **Energy density**  **(Wh/kg)** | **C rate** | **Power density**  **(According to C rate)**  **(W/kg)** | **Specific Capacity**  **(mAh/g)** | **Estimated actual discharge time**  **(h)** | **Actual Power Density**  **(W/kg)** | **References** |
| --- | --- | --- | --- | --- | --- | --- |
| 1.5151 (1.45 Ah) | 0.1 | 15.1 | 1078 | Actual 0.5  Reported | 75.5 | Small 2024, 20, 2311850 |
| 405 (1.2Ah) | 0.1 | 40.5 | ~994.3 | 1.87  (0.53C) | 68.2 | Energy and Environmental Science 2025, 18 (9), 4053–4067. |
| 713 (11.3Ah) | 0.1 | 71.3 | - | - | - | Communications Engineering 2024, 3 (177) |
| 761 (12.0Ah) | 0.05 | 38.05 | - | - | - | Communications Engineering 2024, 3 (177) |
| 325.28 | 0.14 | 16.52 | 1034.19 | 4.41h  (0.23C) | 73.6 | Energy Environ. Sci., 2021,14, 4115-4124 |
| 118 (146mAh) | 0.14 | 16.52 | 730 | 3.11h  (0.32C) | 37.9 | ACS Energy Lett. 2020, 5, 4, 1177–1185 |
| 386.1 (230mAh) | 0.1 | 38.61 | - | - | - | Chem. Commun., 2024,60, 9078-9081 |
| 328 (1.125 Ah) | 0.1 | 32.8 | 1125.2 | 6.7h  (0.15C) | 48.8 | Advanced Materials38, no. 11 (2026): e22467 |
| 137.7 (3.4Ah) | 0.2 | 38.05 | - | - | - | Chemical Engineering Journal,525,  2025,170341 |
| 320 (1.14Ah) | 0.4 | 80 | 953 | 1.42h  (0.7C) | 225.0 | Electrochimica Acta,464,2023,  142889 |
| 103 (390mAh)  (1.35 Ah @0.1C) | 1 | 103 | 326 | 0.19h  (5.1C) | 529.2 | Electrochimica Acta,464,2023,  142889 |
| 317 (1Ah) | 0.02 | 6.34 | 1200 | 35.8h  (0.03) | 8.84 | Nature Nanotechnology 2021, 16 (2), 166–173. |
| 193 (-) | 0.033 | 6.369 | 800 | 14.5h  (0.07C) | 13.3 | ACS Applied Materials and Interfaces 2023, 15 (19), 23104–23114 |
| 434 (3Ah) | 0.025 | 10.85 | 1399 | 33.4h  (0.03C) | 13.0 | Advanced Energy Materials 2023, 13 (42).. |
| 695 (6Ah) | 0.005 | 3.475 | 1563 | 186.6h  (0.005) | 3.72 | Journal of Energy Chemistry 2023, 76, 181–186 |
| 417 (6Ah) | 1.5 | 2766 (reported) | - | - | - | Adv. Mater.2024, 36, 2312880 |
| 190 | 1.5 | 285 | - | - | - | Adv. Mater.2024, 36, 2312880 |
| 250 | 1 | 250 | - | - | - | Adv. Mater.2024, 36, 2312880 |
| 330 (1 Ah) | 0.05 | 16.5 | 1200 | 14.32h  (0.07C) | 23.0 | Adv. Energy Mater.2022, 12, 2202474 |
| 156 (89 mAh) | 0.3 | 46.8 | 938.0 | 1.87h  (0.54C) | 83.6 | Adv. Energy Mater. 2025, 15, 240309 (PVP-I) |
| 215 (314 mAh) | 0.1 | 21.5 | 1144.8 | 6.83h  (0.15C) | 31.5 | Adv. Energy Mater. 2025, 15, 240309 (PVP-I) |
| 140 (209 mAh) | 0.5 | 70 | 789.3 | 0.94h  (1.06C) | 150 | This work |
| 105 (76mAh) | 0.5 | 52.5 | 632.2 | 0.76h  (1.33C) | 140 | This work |
| 86 (57mAh) | 0.5 | 43 | 549.7 | 0.66h  (1.52c) | 132 | This work |

Figure S1. Optimised structures of PVA and PVA-metal (PVA-Cu, PVA-Zn, PVA-Fe, PVA-Ga and PVA-Al complexes.

Two protons are removed from the PVA double layer and replaced with divalent metal cations (Cu^2+^, Zn^2+^) to form charge-neutral divalent PVA-metal complexes. Similarly, three protons are removed to form trivalent PVA complexes with Fe^3+^, Ga^3+^, and Al^3+^.

Figure S2. TEM-EDX elemental mapping of the PVA-Cu binder showing the distribution of Cu, C and O.

Figure S3. Top-view SEM images of sulfur cathodes with each binder system: PVA, PVA-Cu, PVA-Zn, PVA-Fe, PVA-Ga, and PVA-Al.

Figure S4. Nyquist plots of symmetric cells using two identical PVA-Cu and PVA cathodes.

Figure S5: Energy dispersive X-ray (EDX) mapping of PVA-Fe and PVA-Zn cathodes showing homogeneous distribution of Fe and Zn metals in the cathodes.

Figure S6: (a) CV peak currents of the reduction and oxidation peaks and (b) Polarization of CV curves for Li-S batteries using metal-polymer binders at 0.1 mV s^-1^.

The CV results show that the peak current of each catalyst correlates well with the corresponding discharge–charge capacities. Among them, the PVA-Cu cathode shows the highest peak on the volcano plots for both reduction (R2) and oxidation (O) processes, indicating markedly enhanced reaction kinetics in both directions. In contrast, the variation in peak current for *R*_1_ is minimal, whereas substantial differences are observed in the current responses for peaks *R*_2_ and *O*. This suggests that while PVA itself exhibits some degree of LiPS adsorption, it cannot effectively catalyse the rate-limiting redox reactions, likely due to its intrinsically low catalytic activity in the absence of metal components. Furthermore, the polarisation observed in the CV curves exhibits a consistent trend, as observed in the charge-discharge curves (Figure 2c), indicating improved reversibility and faster charge-discharge reactions enabled by the PVA-Cu binder system compared to other PVA-metal binder systems.

Figure S7: Tafel slopes of PVA-Cu, Fe, Zn, GA and Al cathodes corresponding to reduction and oxidation peaks.

Figure S8: Higher sulfur utilization of PVA-metal cathodes than the PVA-only control across C-rates from 0.1 C to 2 C.

Figure S9: (a-d) CV profiles of Li-S cells composed with PVA-metal binders and control PVA system at different scan rates of 0.06 to 0.1 mV s^-1^. (f-h) The linear fits (R^2^> 0.99) of the CV peak currents vs the square root of scan rates at 0.06 to 0.1 mV s^-1^ for the Li-S cells with PVA-metal binders.

Figure S10: Galvanostatic intermittent titration technique analysis of PVA-Cu and PVA cells at 0.1C rate.

Figure S11: Arrhenius relationship between *R*_ct_ and temperature of the PVA-Cu and PVA control cells at different discharge voltages.

Figure S12: Kinetic experiments using Li_2_S_6_ symmetric cells and Li_2_S_8_ cells. (a) CV profiles and (b) Nyquist plots of PVA-metal catalytic binders and PVA control binder system in Li_2_S_6_ symmetric cells (c-d) The Li_2_S dissolution analysis of Li_2_S_8_ cells using PVA-Cu and PVA control binder systems showing potentiostatic discharge of (c) PVA-Cu and (d) PVA control systems.

Figure S13: Ex situ post-mortem top-view SEM images of PVA-Cu and PVA cathodes after 100 cycles of intensive rate cycling from 0.1C to 2C.

Figure S14: Ex situ post-mortem lithium metal anode images of PVA-Cu and PVA based Li- S batteries after 100 cycles of intensive rate cycling from 0.1C to 2C.

Figure S15: EDX analysis of ex-situ post-mortem lithium metal anode coupling with PVA-Cu after 100 cycles of rate capability testing from 0.1C to 2C.

Figure S16: (a) Comparison of the XPS spectra of Li metal anodes recovered from cells using PVA and PVA-Cu cathodes after cycling (after 100 cycles of rate capability testing from 0.1C to 2C). The atomic percentages of S2p, O1s, F1s, C1s, and N1s are summarized in Table S5. (b) XPS Cu 2p spectra of the Li metal anode from the PVA-Cu cell, confirming the absence of Cu on the Li metal anode.

Figure S17: 13 mg cm^-2^ sulfur loading PVA-Cu cathode showing high areal capacity of 14.7 mAh cm^-2^.

Figure S18: Linear increase in areal capacity as a function of sulfur loading at (a) 0.1C and (b) 0.5C rates of the PVA-Cu catalytic binder system.

Figure S19: Comparing the discharge and areal capacity of PVA-Cu cathodes with recently published literature after 200 cycles at a 1C rate [4-14].

Figure S20: Electrochemical performance between Cu-polymer complexes and Cu and CuO nanoparticles. (a) Galvanostatic charge-discharge profiles and (b) corresponding capacities delivered at first discharge plateau (*R*_1_), second discharge plateau (*R*_2_) and oxidation (*O*). (c) CV profiles and (d) corresponding CV peak currents delivered at *R*_1_, *R*_2_ and *O*. (e) *R*_ct_ values in EIS plots of Cu-polymer complexes (PVA-Cu and PVP-Cu) and Cu based nanoparticles.

Figure S21: Optimized adsorption configurations of Li_2_S_6_ on PVA and PVA-metal complexes (PVA-Cu, PVA-Zn, PVA-Fe, PVA-Ga, and PVA-Al).

Figure S22: Optimized adsorption configurations of Li_2_S_6_ on PVA and PVA-metal complexes (PVA-Cu, PVA-Zn, PVA-Fe, PVA-Ga, and PVA-Al).

Figure S23: PVA-Cu pouch cells characterisation. a) Rate capability from 0.1C to 0.5C rate and long-term cycling at 0.5C rate b) The charge-discharge profiles of 1^st^ and 100^th^ cycle at 0.5C pouch cell.

Figure S24: Statistical analysis of Coulombic efficiency (CE) obtained from three independently assembled Li-S cells under different sulfur loadings and cycling rates.

PVA-Cu cells with sulfur loadings of 1.5-3.0 mg cm^-2^ cycled at 2C-3C maintain an average Coulombic efficiency (CE) above 99.5% over 200 cycles. Furthermore, for cells with a sulfur loading of 3.0 mg cm-2 cycled at 1C, the average CE remains above 99% during the first 100 cycles and gradually decreases to approximately 98.5% after 200 cycles. This slight decrease is consistent with the behavior commonly observed in Li-S batteries during extended cycling, in which increased polysulfide concentration and parasitic side reactions at the Li-metal anode can gradually reduce CE.

Figure S25: XPS spectra of metal 2p regions for PVA-metal coordination complexes. High-resolution XPS spectra of (a) Cu 2p, (b) Fe 2p, (c) Zn 2p, (d) Ga 2p, and (e) Al 2p regions confirming the oxidation states of the corresponding metal centers in the PVA-metal complexes.

The XPS spectra confirm the expected oxidation states of Cu^2+^, Fe^3+^, Zn^2+^, Ga^3+^ and Al^3+^ in the corresponding polymer-metal systems. Specifically, PVA-Cu exhibits Cu 2p_3/2_ and Cu 2p_1/2_ peaks at 934.5 and 954.4 eV, accompanied by satellite peaks at 944.5 and 962.4 eV, confirming the presence of Cu^2+^ [15, 16]. Fe 2p peaks at 711 and 724.3 eV [15, 17], Zn 2p peaks at 1022 and 1045.1 eV[15, 18], Ga 2p peaks at 1118.1 and 1144.9 eV [19], and Al 2p at 74.88 eV [18] confirm the Fe^3+^, Zn^2+^, Ga^3+^ and Al^3+^ oxidation states, respectively.

In response, we have included the full cycling profiles in the Supporting Information, as shown in Figure S26.

Figure S26: Complete cycling profiles, including the initial activation cycles, of PVA-Cu cathodes with sulfur loadings of (a) 4 mg cm^-2^ and (b) 6 mg cm^-2^ at 1C rate and (c) 7 and 9 mg cm^-2^ at 0.5 C rate.

***Explanatory Note 1***: For quantitative analysis, time and current are normalised by t_max_ and I_max_, respectively, to compare with four classical electrochemical deposition models. Among these, two-dimensional progressive (2DP) and two-dimensional transient (2DI) nucleation are controlled by the incorporation of atoms at the lattice interface. Three-dimensional progressive (3DP) and three-dimensional transient (3DI) nucleation are achieved through volume-diffusion-controlled growth.

2D nucleation can be fitted through the A. Beweick, M. Fleischman, and H.R. Thirsk (BFT) models and 3D nucleation can be fitted through the Scharifker-Hills (SH) models. (𝑗_𝑚_ corresponds to the current density, and 𝑡_𝑚_ corresponds to the time at the point of maximum current density).

The PITT curves obtained for both PVA-Cu and PVA cathodes were fitted to the four nucleation models: 2DP, 2DI, 3DP, and 3DI. The goodness of fit was evaluated using the coefficient of determination (R^2^).

For the PVA-Cu cathode, the 3DP model exhibited the highest R^2^ value of 0.90, indicating that Li_2_S deposition predominantly follows a three-dimensional progressive nucleation-and-growth mechanism. In contrast, for the PVA cathode, the highest R^2^ value of 0.78 was obtained with the 2DI model, suggesting a less favorable nucleation process characterized by mixed or predominantly two-dimensional instantaneous growth behavior.

*Table S12: Statistical comparison of 2DI, 2DP, 3DI, and 3DP nucleation models based on R^2^*

*values obtained from fitting experimental PITT data.*

| *Models* | *R^2^ of PVA* | *R^2^ of PVA-Cu* |
| --- | --- | --- |
| *3DI* | *0.34* | *0.52* |
| *3DP* | *0.64* | ***0.90*** |
| *2DI* | ***0.78*** | *-0.98* |
| *2DP* | *-1.97* | *-10.46* |

**References**

[1] Beuse, T.; Fingerle, M.; Wagner, C.; Winter, M.; Börner, M. Comprehensive Insights into the Porosity of Lithium-Ion Battery Electrodes: A Comparative Study on Positive Electrodes Based on LiNi0.6Mn0.2Co0.2O2 (NMC622). *Batteries* 2021, *7* (4), 70. DOI: 10.3390/batteries7040070

[2] Wang, N.; Zhang, X.; Ju, Z.; Yu, X.; Wang, Y.; Du, Y.; Bai, Z.; Dou, S.; Yu, G. Thickness-independent scalable high-performance Li-S batteries with high areal sulfur loading via electron-enriched carbon framework. *Nature Communications* 2021, *12* (1). DOI: 10.1038/s41467-021-24873-4

[3] Jovanović, P.; Shaibani, M.; Nishshanke, M. M.; Zavabeti, A.; Hill, M. R.; Majumder, M. Noncollapsible, Meringue-Structured Cathodes for a Volumetrically Efficient Li–S Battery. *Small Structures* 2025. DOI: 10.1002/sstr.202500537

[4] Li, Z.; Sami, I.; Yang, J.; Li, J.; Kumar, R. V.; Chhowalla, M. Lithiated metallic molybdenum disulfide nanosheets for high-performance lithium–sulfur batteries. *Nature Energy* 2023, *8* (1), 84–93. DOI: 10.1038/s41560-022-01175-7

[5] Yang, R.; Chen, Y.; Pan, Y.; Kim, M.; Liu, H.; Lee, C. K. W.; Huang, Y.; Tang, A.; Tu, F.; Li, T.; et al. Single-step laser-printed integrated sulfur cathode toward high-performance lithium–sulfur batteries. *Nature Communications* 2025, *16* (1). DOI: 10.1038/s41467-025-57755-0

[6] Wu, Z.; Liu, M.; He, W.; Guo, T.; Tong, W.; Kan, E.; Ouyang, X.; Qiao, F.; Wang, J.; Sun, X.; et al. Unveiling the autocatalytic growth of Li2S crystals at the solid-liquid interface in lithium-sulfur batteries. *Nature communications* 2024, *15* (1), 9535–9535. DOI: 10.1038/s41467-024-53797-y

[7] Li, H.; Meng, R.; Ye, C.; Tadich, A.; Hua, W.; Gu, Q.; Johannessen, B.; Chen, X.; Davey, K.; Qiao, S. Z. Developing high-power Li||S batteries via transition metal/carbon nanocomposite electrocatalyst engineering. *Nature Nanotechnology* 2024, *19* (6), 792–799. DOI: 10.1038/s41565-024-01614-4

[8] Lv, S.; Ma, X.; Ke, S.; Wang, Y.; Ma, T.; Yuan, S.; Jin, Z.; Zuo, J. L. Metal-Coordinated Covalent Organic Frameworks as Advanced Bifunctional Hosts for Both Sulfur Cathodes and Lithium Anodes in Lithium-Sulfur Batteries. *Journal of the American Chemical Society* 2024, *146* (13), 9385–9394. DOI: 10.1021/jacs.4c01620

[9] Chen, J. Z.; Li, Z. A.; Lei, J. T.; Chen, P. P.; Zhao, D. L. Accelerated Ion-Electron Transport in Bi-Heterostructures Constructed Based on Ohmic Contacts for Efficient Bi-Directional Catalysis of Lithium-Sulfur Batteries. *Small* 2024. DOI: 10.1002/smll.202408284

[10] Ji, L.; Yang, D.; Xue, J.; Jia, M.; Wu, T.; Zhuang, Q.; Zhang, Y.; Liu, J.; Zhang, Y. Flexible Titanium Nitride-Based Membrane Reactor for S8/Li2S and Dendrite Regulation in Lithium-Sulfur Batteries. *Advanced Energy Materials* 2025. DOI: 10.1002/aenm.202404738

[11] Yu, Z.; Huang, X.; Zheng, M.; Zhang, S. Q.; Yang, Y.; Lu, J. Self-Assembled Macrocyclic Copper Complex Enables Homogeneous Catalysis for High-Loading Lithium–Sulfur Batteries. *Advanced Materials* 2023, *35* (26). DOI: 10.1002/adma.202300861

[12] Hu, S.; Huang, X.; Zhang, L.; Li, G.; Chen, S.; Zhang, J.; Liu, X. Vacancy-Defect Topological Insulators Bi2Te3−x Embedded in N and B Co-Doped 1D Carbon Nanorods Using Ionic Liquid Dopants for Kinetics-Enhanced Li–S Batteries. *Advanced Functional Materials* 2023, *33* (20). DOI: 10.1002/adfm.202214161

[13] Ren, L.; Liu, J.; Zhao, Y.; Wang, Y.; Lu, X.; Zhou, M.; Zhang, G.; Liu, W.; Xu, H.; Sun, X. Regulating Electronic Structure of Fe–N4 Single Atomic Catalyst via Neighboring Sulfur Doping for High Performance Lithium–Sulfur Batteries. *Advanced Functional Materials* 2023, *33* (12). DOI: 10.1002/adfm.202210509

[14] Nishshanke, M. M.; Jovanović, P.; Panda, M. R.; Abedin, M. J.; McNamara, D.; Hill, M. R.; Bhattacharya, J.; Kamal, C.; Shaibani, M.; Majumder, M. Role of Polymer-Iodine Complexes on Solid-Liquid Polysulfide Phase Transitions and Rate Capability of Lithium Sulfur Batteries. *Advanced Energy Materials* 2024. DOI: 10.1002/aenm.202403092

[15] Islam, M. S.; Chowdhury, K. R.; Hoque, S. M.; Sharif, A. Cationic and oxygen defect modulation for tailoring the bandgap and room temperature ferromagnetism of CuO via multiple d-block cations. *Materials Advances* 2024, *5* (7), 2946–2967. DOI: 10.1039/d3ma00987d

[16] Bose, S.; Seonho, J.; Kang, H. W. Biocompatible and hydrophilic copper-complexed polyvinyl alcohol coating for antifogging surfaces. *Polymer Testing* 2024, *140*. DOI: 10.1016/j.polymertesting.2024.108619

[17] Mukherjee, P.; Mandal, M.; Mukherjee, B.; Dutta, G. Nonenzymatic Electrochemical Sensor Device for On-Site Nitrate Determination Using Copper Anchored Magnetite Nanocomposites. *Langmuir* 2025, *41* (10), 6747–6758. DOI: 10.1021/acs.langmuir.4c04903

[18] Moreno, M.; Devis, S.; Velasquez, L.; Arredondo, M.; Mariotti, D.; McGlynn, R.; Sanchéz-Ruderisch, H.; Quijada, R.; González, G.; Olguin, Y.; et al. Zn and Al vapour-phase metalation of polyamide-6 via ALD: structural, functional, and biomedical insights. *Journal of Materials Science* 2025, *60* (44), 22203–22233. DOI: 10.1007/s10853-025-11168-y

[19] Mallesham, B.; Roy, S.; Bose, S.; Nair, A. N.; Sreenivasan, S.; Shutthanandan, V.; Ramana, C. V. Crystal Chemistry, Band-Gap Red Shift, and Electrocatalytic Activity of Iron-Doped Gallium Oxide Ceramics. *ACS Omega* 2020, *5* (1), 104–112. DOI: 10.1021/acsomega.9b01604
